# Evidence of an Exponential Decay Pattern of the Hepatitis Delta Virus Evolution Rate and Fluctuations in Quasispecies Complexity in Long-Term Studies of Chronic Delta Infection

**DOI:** 10.1371/journal.pone.0158557

**Published:** 2016-06-30

**Authors:** Maria Homs, Francisco Rodriguez-Frias, Josep Gregori, Alicia Ruiz, Pilar Reimundo, Rosario Casillas, David Tabernero, Cristina Godoy, Salma Barakat, Josep Quer, Mar Riveiro-Barciela, Michael Roggendorf, Rafael Esteban, Maria Buti

**Affiliations:** 1 Centro de Investigación Biomédica en Red de Enfermedades Hepáticas y Digestivas (CIBERehd), Instituto de Salud Carlos III, Barcelona, Spain; 2 Liver Pathology Unit, Departments of Biochemistry and Microbiology, Hospital Universitari Vall d'Hebron, Universitat Autònoma de Barcelona (UAB), Barcelona, Spain; 3 Liver Diseases Unit, Vall d’Hebron Research Institute, Barcelona, Spain; 4 Roche Diagnostics SL, Sant Cugat del Vallès, Spain; 5 Gastroenterology Department, National Centre for Gastrointestinal and Liver disease, Khartoum, Sudan; 6 Liver Unit, Hospital Universitari Vall d'Hebron, Universitat Autònoma de Barcelona (UAB), Barcelona, Spain; 7 Institut of Virology, Technische Universität München/Helmholtz Zentrum München, Munich, Germany; Hannover Medical School, GERMANY

## Abstract

Chronic HDV infection can cause a severe form of viral hepatitis for which there is no specific treatment. Characterization of the hepatitis B or C viral quasispecies has provided insight into treatment failure and disease recurrence following liver transplantation, has proven useful to understand hepatitis B e antigen seroconversion, and has helped to predict whether hepatitis C infection will resolve or become chronic. It is likely that characterization of the hepatitis delta virus (HDV) quasispecies will ultimately have similar value for the management of this infection. This study sought to determine the RNA evolution rates in serum of chronic hepatitis delta (CHD) treatment-naïve patients, using next-generation sequencing methods. The region selected for study encompassed nucleotide positions 910 to 1270 of the genome and included the amber/W codon. Amber/W is a substrate of the editing process by the ADAR1 host enzyme and is essential for encoding the 2 delta antigens (HDAg). The amber codon encodes the small (unedited) HDAg form and the W codon the large (edited) HDAg form. The evolution rate was analyzed taking into account the time elapsed between samples, the percentage of unedited and edited genomes, and the complexity of the viral population. The longitudinal studies included 29 sequential samples from CHD patients followed up for a mean of 11.5 years. In total, 121,116 sequences were analyzed. The HDV evolution rate ranged from 9.5x10^-3^ to 1.2x10^-3^ substitutions/site/year and showed a negative correlation with the time elapsed between samples (p<0.05). An accumulation of transition-type changes was found to be responsible for higher evolution rates. The percentages of unedited and edited genomes and the quasispecies complexity showed no relationships with the evolution rate, but the fluctuations in the percentages of genomes and in complexity suggest continuous adaptation of HDV to the host conditions.

## Introduction

More than 10 million individuals worldwide are estimated to be carriers of the hepatitis delta virus (HDV) [1], which has the potential to cause a severe form of hepatitis infection [2]. HDV is a unique virus closely related to viroids [3] that requires the hepatitis B virus (HBV) surface antigen (HBsAg) to create its viral envelope. HDV infection causes numerous clinical manifestations, and the risk of developing liver-related complications is higher in patients with HBV/HDV coinfection than in those with HBV infection alone [4].

The delta genome is comprised of a single-stranded circular RNA molecule, approximately 1700 nucleotides in length, whose composition enables 70% internal base pairing and adopts a stable rod-like structure. HDV replicates by a double-rolling-circle model, which involves the host RNA polymerase II and generates a complete complementary copy of the genome, known as the antigenome [5]. In contrast to what occurs in viroids, which have no known coding capacity, HDV has several open reading frames (ORF), but only one is transcribed [6]. This single transcribed ORF can encode 2 hepatitis delta antigens (HDAg), the small (S-DAg) and large (L-DAg) forms [3]. L-DAg is identical to the small antigen except for a short C-terminal extension, arising due to a single post-transcriptional editing mechanism involving the host RNA-specific adenosine deaminase, ADAR1. Editing leads to an A to G change at nucleotide 1415 of the viral antigenome (reference sequence [7]). Position 1415 of the antigenome corresponds to position 1014 of the genome and to codon 196 of the HDAg ORF (amber/W codon).

Unedited genomes carry the 5’-CUA sequence at positions 1013 to 1015, which is transcribed to mRNA as 5’-UAG, is translated into mRNA as an amber stop codon at position 196, and encodes S-DAg. In contrast, edited genomes carry the 5’-CCA sequence at positions 1013 to 1015, which is transcribed to mRNA as 5’-UGG and translated into tryptophan at codon 196, maintaining translation to a second UAG codon located in position 215, and encoding L-DAg [8]. The presence of this tryptophan enlarges the antigen with an additional 19 to 20 amino acids, and results in several degrees of functionality. S-DAg supports HDV replication, whereas L-DAg, which contains a prenylation Caxx box, interacts with HBsAg to assemble new HDV virions [9]. This prenylation box represents a potential therapeutic target for treating HDV infection [10].

HDV circulates as a “cloud” of virions that are different from each other but highly related, known as a quasispecies [11,12]. Study of viral quasispecies has been traditionally based on cloning and sequencing, and more recently on a more sensitive and reproducible method known as next-generation sequencing (NGS) [13–15]. In HDV, this method has been used in samples derived from cell cultures [12], but never in clinical samples. The RNA evolution rate, defined as the rate at which RNA substitutions occur, is a useful parameter to understand viral adaptation to the host. The HDAg ORF has been estimated to have 1.4x10^-2^ to 1.5x10^-3^ substitutions/site/year [16], but this rate has not been confirmed in long-term studies of chronic delta infection, or by deep sequencing. Another way to characterize the HDV quasispecies is to determine its complexity (ie, the number of different virions comprising it) and the dynamics of this complexity (ie, the changes that occur in the quasispecies composition) through the use of various diversity indices, as has been done in infections caused by HBV and hepatitis C virus (HCV) [14,17].

The aim of this study was to evaluate RNA evolution in the HDV quasispecies by NGS in serum samples from patients with CHD infection, and to study the relationships of evolution with the type of changes accumulated in the genome, the complexity of the HDV quasispecies, and the percentage of each codon at the amber/W codon position. For this purpose, a selected region of the HDV genome including the amber/W codon was analyzed in 3 long-term sequential studies.

## Materials and Methods

### Materials

**Fig 1:**
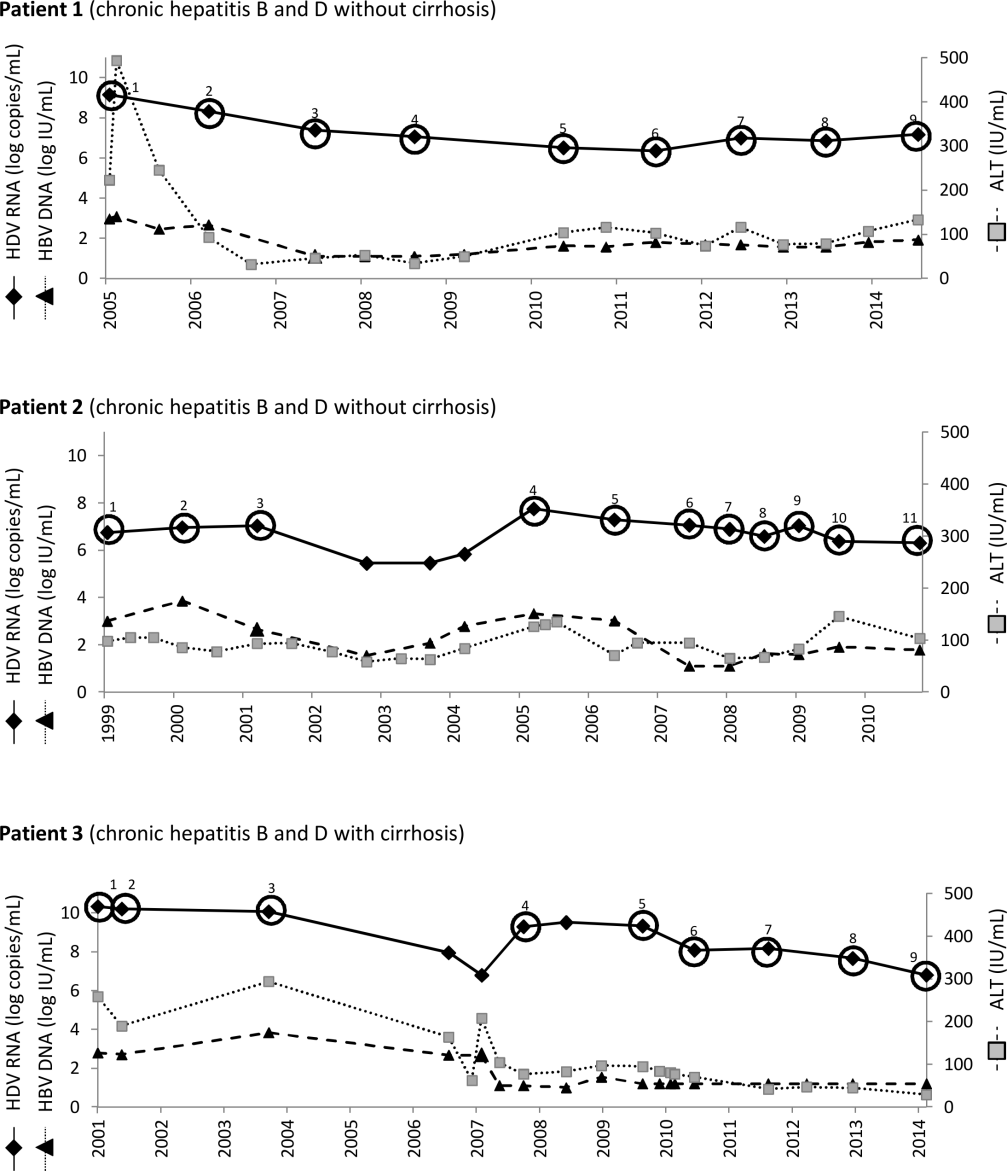
Serum HDV RNA, HBV DNA, and ALT levels in the long-term follow-up studies of 3 patients. Samples selected for evaluation of the HDV quasispecies and RNA changes are indicated by circles and numbers.

The Ethics Committee of Vall d’Hebron Research Institute approved this retrospective study, which includes sequential samples from 3 patients with CHD infection, who gave written consent for participation. The selection criteria were treatment-naïve status, low HBV DNA levels, similar HDV RNA levels between patients, and a lengthy follow-up without antiviral treatment. Twenty-nine samples from 3 untreated chronic HDV patients with a mean follow-up of 11.5 years (SD, 1.8) were selected to determine RNA evolution. The sequential samples selected for analysis and the patients’ HDV RNA, HBV DNA, and ALT levels over the long-term study are shown in Fig 1.

### Region of HDV genome analyzed by NGS

The region selected for analysis covered nucleotide positions 910 to 1270 of the HDV genome and included the amber/W codon of the HDAg ORF. Molecular amplification and deep sequencing of the HDV genome was done on the 454 GS Junior system (454 Sequencing Systems, Roche Diagnostics), which enables analysis of fragments up to 500-bp in length. A detailed description of the procedures and subsequent data processing are provided in the Supplementary Materials and Methods (S1 Protocol).

Briefly, the sequencing data were demultiplexed and primers were trimmed. After a quality filter step, sequences covering the full amplicon and common to both strands were selected. Haplotypes, that is, unique sequences covering the full amplicon observed on the clean set of sequences, were identified [18]. All computations were done in the R environment and language, and were based on an in-house-developed pipeline [15,19,20]. The NGS sequencing data from the samples analyzed have been submitted to the NCBI Sequence Read Archive (SRA) database. The BioProject accession number is PRJNA308541. The BioSample accession numbers are included in S1 Table.

### Evolution rates in the HDV RNA sequences

The evolution rate was assessed as the frequency of substitutions detected in each sequential sample in comparison with the most highly represented haplotype in the first sample from each patient (as the first sample was taken as the reference to calculate the corresponding evolution rate, evolution was assessed in 26 of the 29 samples). Haplotype frequencies were considered a surrogate measure of mutation fixation and are expressed relative to the length of the region and the time elapsed between samples.

In addition, the evolution rate was assessed separately for sequences carrying either amber or W at the amber/W codon. In these analyses, the edited and unedited haplotypes were compared with the most frequent unedited and edited haplotypes present in the first sample. As the 2 variants might have different life cycles, the evolution rate was also computed separately for each of them for the complete region without the editing codon, thus allowing to calculate global evolution rate avoiding biases due to editing codon.

### Accumulation of mutations and types of changes in the HDV RNA sequences

The accumulation of mutations was calculated by recording all the nucleotide changes observed in the sequential studies (haplotype frequencies not taken into account), in comparison with the dominant haplotype sequence from the first sample of each patient (26 of the 29 samples analyzed). The accumulation of mutations was assessed for the complete region and for the 2 different genomes.

### Percentage of unedited and edited genomes

The percentage of haplotypes per sample with CUA at positions 1013–1015 of the genome (translated into the UAG codon at 196) determined the percentage of unedited genomes (encoding S-HDAg). The percentage of haplotypes per sample with CCA (translated into the UGG codon in 196) at positions 1013–1015 of the genome determined the percentage of edited genomes (encoding L-HDAg). The percentages of unedited and edited genomes were calculated for the 29 sequential samples.

### HDV quasispecies complexity

Six parameters were used to describe the HDV quasispecies: the number of haplotypes and the number of mutations as incidence-based indices, the Hill numbers of order (q = 1 and q = 2) as abundance-based indices, and the mutation frequency and nucleotide diversity as functional indices [21,22]. A detailed description of the meaning of each parameter is described in Supplementary Materials and Methods (S1 Protocol). These indices were determined in the 29 sequential samples. To compare the quasispecies complexity between groups of samples, a down-sampling plus fringe-trimming approach was applied before computing the diversity indices [18,19]. In addition, changes occurring in the parameters describing complexity of quasispecies were assessed in each patient over follow-up using multidimensional data analyses. A detailed explanation of these analyses is provided in S1 Protocol.

### Routine methods

HBV DNA was quantified by the COBAS TaqMan HBV test V2.0 (Roche Diagnostics, GmbH, Mannheim, Germany) and HDV RNA was quantified by an in-house method, based on a complete genomic HDV RNA standard [23]. HDV genotyping was performed by Sanger sequencing. The region analyzed by NGS was also used for genotyping. Both methods identified HDV as genotype 1 in all samples.

### Statistical analysis

Statistical analyses were carried out with IBM SPSS 20 (SPSS Inc., Chicago, USA). Categorical data were tested using a *t*-test for paired samples and non-categorical data using the Wilcoxon test for paired samples. Correlations were evaluated with the Pearson test. Significance was set at p≤0.05.

## Results

### Evolution rate of HDV RNA sequences

A total of 242,081 raw reads, yielding 121,116 quality-filtered sequences (mean 4176 sequences/sample, SD 2543), were obtained in the long-term sequential studies (S2 Table). The HDV genome region from nucleotide 910 to 1720 yielded a mean evolution rate of 2.8x10^-3^ substitutions/site/year (SD, 2.1x10^-3^). The evolution rate for each patient showed similar mean values (patient 1, 2.5x10^-3^ substitutions/site/year, patient 2, 2.4x10^-3^ substitutions/site/year and patient 3, 3.7x10^-3^ substitutions/site/year), but the comparison between patients yielded a significant difference (p = 0.031). This difference was mainly due to the results in patient 3, with high evolution values in the 2 initial samples.

**Fig 2:**
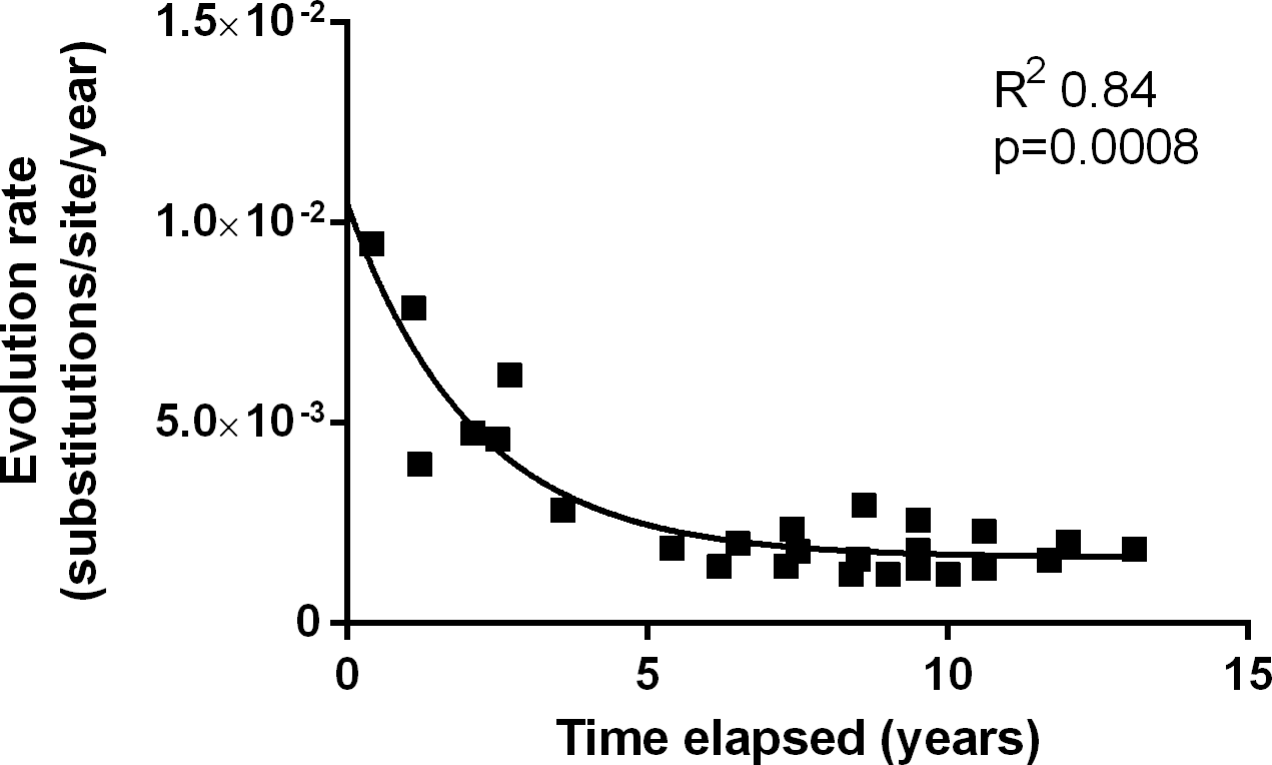
Exponential decay pattern of the evolution rate in relation to the time elapsed between samples.

The overall evolution rate ranged from 1.2x10^-3^ to 9.5x10^-3^ substitutions/site/year. This range showed a significant exponential decay with the time elapsed between samples (Fig 2), indicating that the evolution rate has 2 phases: an initial decrease and a second stable phase. This pattern was also observed separately in the sequential studies (S1 Fig). In addition, the evolution rate positively correlated with HDV RNA levels (R^2^ = 0.23, p = 0.013). However, when the 3 patients were analyzed independently, a significant correlation was only found in patient 3 (R^2^ = 0.54, p = 0.036).

**Fig 3:**
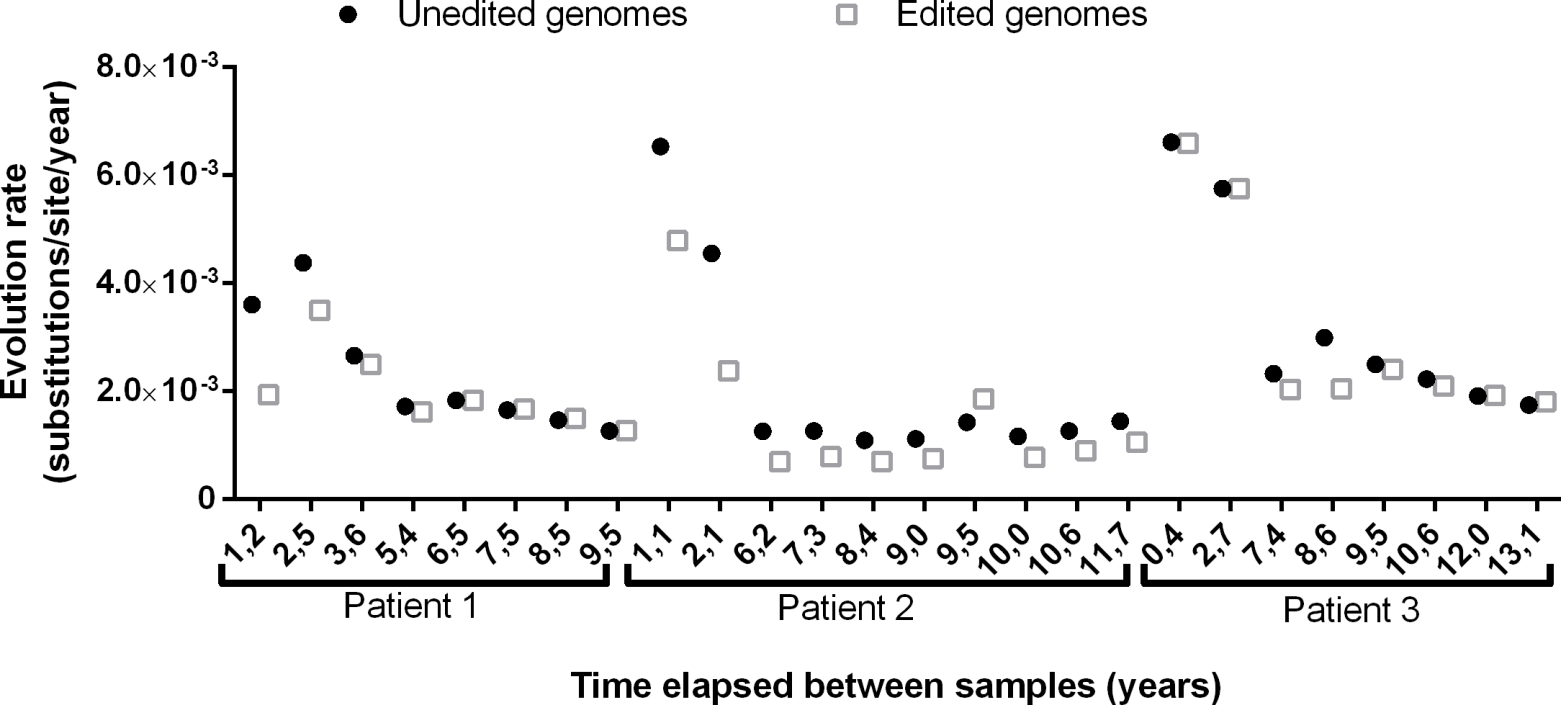
Evolution rate of the unedited and edited genomes in relation to the time elapsed, for the 3 long-term studies.

The method and filtering developed in this study enabled analysis of the genomes in the circulating HDV quasispecies, differentiated by the type of codon at amber/W. To investigate differences between genomes encoding S-HDAg (amber codon) and those encoding L-HDAg (W codon), the evolution rates of these genomes were calculated separately in each patient (Table 1). Mean evolution values were similar between genomes encoding the 2 HDAg forms, but paired analysis of all samples showed a significant difference between them (p = 0.001): in 18 of 26 samples the evolution rate was higher in genomes with the amber codon than in those with the W codon (Fig 3). The evolution of the 2 genomes also showed an exponential decay associated with the time elapsed between sequential samples (S2 Fig).

**Table 1:** Evolution rate of the unedited (amber codon) and edited (W codon) genomes, observed in the 3 cases studied.

Patients	Evolution rate of unedited genomes (substitutions/site/year)	Evolution rate of edited genomes (substitutions/site/year)	p-value
	Mean (SD)	Mean (SD)	
1	2.3x10^-3^ (1.1 x10^-3^)	2.0 x10^-3^ (1.3 x10^-3^)	
2	2.1 x10^-3^ (1.8 x10^-3^)	1.5 x10^-3^ (1.9 x10^-3^)	0.001
3	3,3 x10^-3^ (1.8 x10^-3^)	3.1 x10^-3^ (1.9 x10^-3^)	

To understand this decay pattern, we investigated the types of nucleotide changes accumulated in the HDV RNA sequences, the changes in the percentage of unedited and edited genomes, and the dynamics of HDV quasispecies complexity.

### Types of nucleotide changes in HDV RNA sequences

All types of nucleotide changes were computed in the sequential samples. Overall, nucleotide transitions accounted for a mean of 77.02% (SD, 9.35%) of changes. Transitions were more frequent than transversions (mean, 22.98% of changes, SD 9.35%, p<0.001) in the complete region analyzed (S3 Table, panel a). Even after excluding the amber/W codon (S3 Table, panel b), which is a hot spot for A to G changes in the antigenomic form, transitions (mean 74.2%, SD 10.5) were more common than transversions (mean 25.8%, SD 10.05) (p<0.001). Interestingly, among the 4 possible transition-type changes, C to U and U to C accounted for a mean of 94.22% (SD, 7.78), whereas G to A and A to G represented only 5.78% (SD, 7.78) (p<0.001). On analysis of unedited and edited genomes separately (S3 Table, panels c and d), nucleotide transitions were also more frequent than nucleotide transversions in both types (p<0.001), and U to C and C to U were significantly more common than A to G or G to A changes (p<0.001). However, U to C changes were significantly more frequent in unedited than edited genomes (p = 0.009), indicating that the type of nucleotide accumulated differs between the 2 genomes.

**Fig 4:**
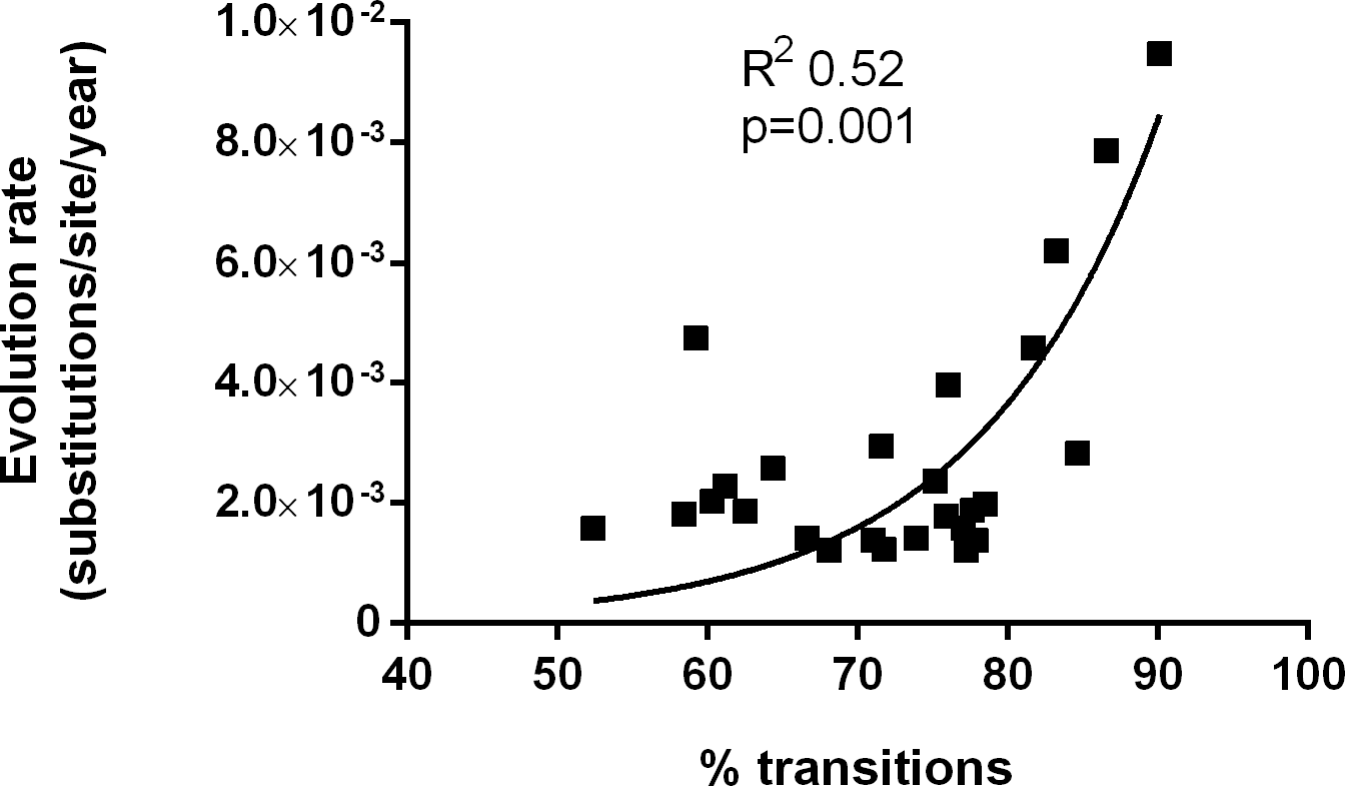
Exponential growth pattern between the evolution rate and the percentage of transitions accumulated in HDV genome.

To determine whether the nucleotide changes were associated with the evolution pattern, a correlation study was performed between the evolution rate values and the most significant nucleotide changes (percentage of transitions with respect to the overall changes, and percentage of C to U and U to C changes with respect to the overall changes and with respect to the transitions). An exponential growth relationship was observed between the evolution rate and the percentage of transitions (Fig 4), indicating that this type of nucleotide change was responsible for the higher evolution rates in the 3 sequential studies.

### Dynamics of unedited and edited HDV genomes

**Fig 5:**
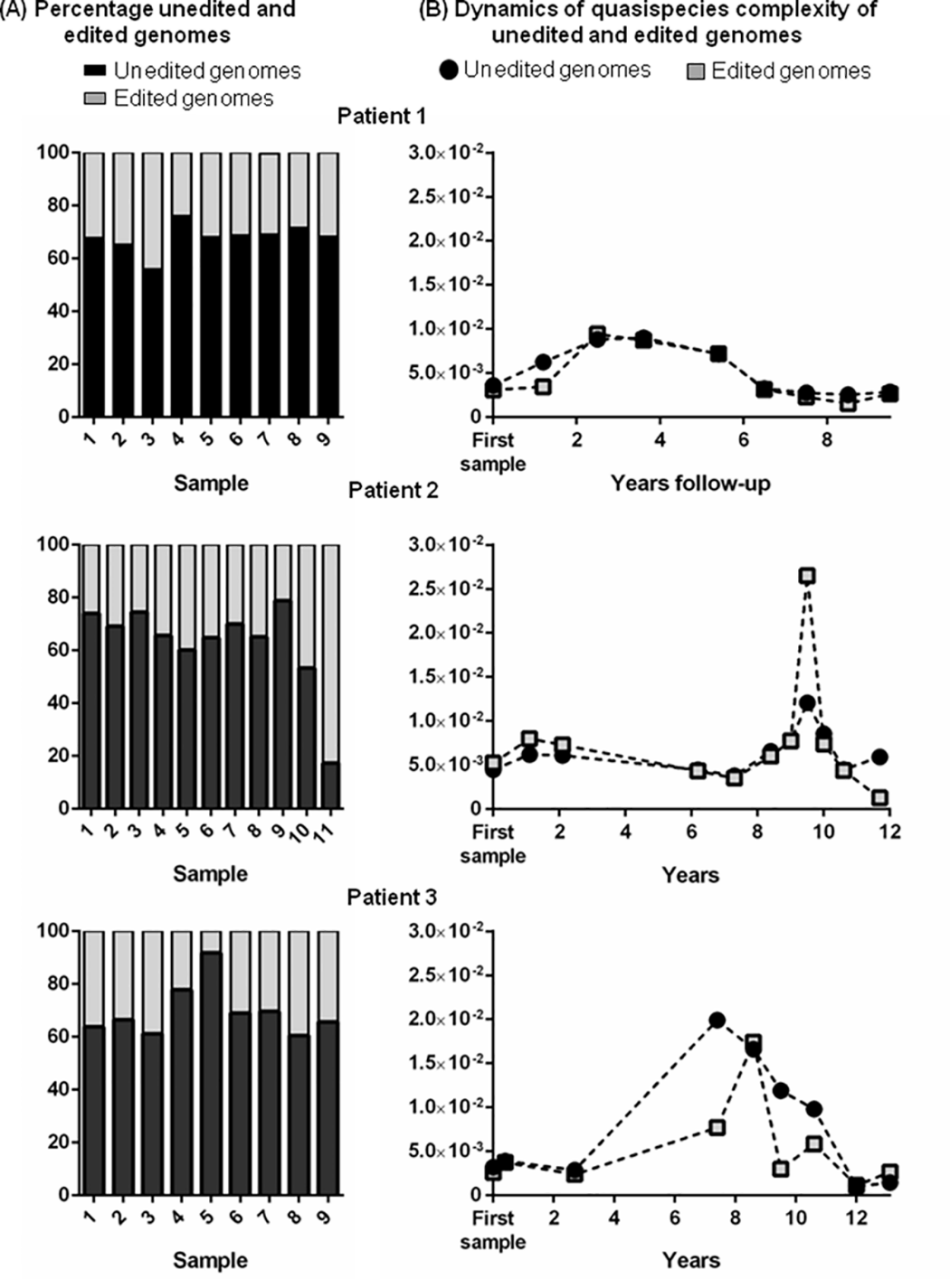
**(A) Changes in the percentage of unedited and edited genomes (B) Quasispecies complexity dynamics (nucleotide diversity) in the long-term follow-up**.

As was mentioned above, the technique and filter developed in the present study enable genome classification according to the codon at the amber/W position. In the 29 samples, this codon translated only to tryptophan (edited) and to stop codons (unedited); no other amino acids were detected at this position. In the overall sequential samples, 66.5% (SD, 12) of genomes were unedited and 33.5% were edited (SD, 12). In 28 of the 29 samples, unedited genomes predominated over edited ones. The percentage of unedited and edited genomes fluctuated over the long-term studies (Fig 5, panel A), but no relationship was observed between these percentages and the evolution rate.

### Dynamics of HDV quasispecies complexity

The quasispecies complexity in the region analyzed was evaluated using 6 parameters (Table 2). As described above, 66.5% of genomes had amber codons and 33.5% W codons at the amber/W codon. Because the percentages of these codons could be an artifact when assessing quasispecies complexity, we performed an analysis excluding the W/amber codon and found significantly lower quasispecies complexity values than those recorded for the complete region (Table 2). In the 2 genomes studied, quasispecies complexity was assessed by mutation frequency and nucleotide diversity (2 parameters that are virtually unaffected by bias related to the number of sequences analyzed [19]). Quasispecies complexity showed a parallel fluctuation in the 2 genomes over the 3 sequential studies (Fig 5, panel B), and the quasispecies complexity of unedited genomes was similar to that of edited genomes in a paired-samples analysis (p = 0.16). Inverse dynamics, with an increase in unedited genomes and a decrease in edited genomes, were seen in only 3 samples (sample 11, patient 2, and samples 5 and 7, patient 3).

**Table 2:** Mean and standard deviation of the indices of quasispecies complexity obtained in the complete region and after excluding the amber/W codon.

Quasispecies complexity indices	Complete region (N = 29)	Amber/W codon excluded (N = 29)	p-value
	Mean (SD)	Mean (SD)	
Number of haplotypes	46.20 (22.19)	34.07 (18.73)	<0.001
Number of mutations	18.34 (10.94)	17.52 (10.61)	<0.001
Hill order number q = 1	19.27 (14.70)	12.45 (10.81)	<0.001
Hill order number q = 2	10.56 (8.52)	6.78 (6.20)	<0.001
Mutation frequency	4.8x10^-3^ (3.0x10^-3^)	3.8x10^-3^ (2.9x10^-3^)	<0.001
Nucleotide diversity	7.0x10^-3^ (4.1x10^-3^)	5.8x10^-3^ (4.0x10^-3^)	<0.001

Associations between the evolution rate and the quasispecies complexity were investigated using the parameters obtained after excluding the W/amber codon. No relationships were found between the evolution rate and quasispecies complexity indices. The complexity values obtained did not correlate with the percentage of unedited and edited genomes or with HDV RNA level.

The dynamics of quasispecies complexity over follow-up in the 3 patients analyzed showed different periods of increasing and decreasing complexity. The maximum complexity peaks did not show a common pattern, assessed by Principal Component 1 (PC1, S3 Fig and S1 Protocol): patient 1, between 2 and 4 years; patient 2, between 8 and 10 years; and patient 3, between 6 and 8 years.

## Discussion

The HDV quasispecies in serum samples of patients with chronic delta infection has never been assessed by NGS, currently the most reliable and reproducible method available for this purpose [15,19]. In previous studies, we performed control reactions with clones and repeated NGS experiments in different laboratories to assure the reproducibility of the technique for investigating viral hepatitis quasispecies [15,19]. The sequencing data filter, the down-sampling and fringe-trimming approach, and the selection of the quasispecies complexity indices used in the present study assure reliable evaluation and comparison of HDV.

The evolution rate in the HDAg ORF determined by cloning has been estimated at 1.4x10^-2^ to 1.5x10^-3^ substitutions/site/year [16]. Higher evolution rates have been associated with the acute hepatitis phase and lower rates with the chronic state. Previous NGS assessment of HDV evolution has been done in a single *in vitro* study focused on the right terminal domain of the HDV genome, with results ranging from 2.9x10^-3^ to 0.8x10^-3^ substitutions/site/year [12]. In the present study we evaluated a 360-bp region that included part of the HDAg ORF, which was also used for genotyping. Evolution in the complete region ranged from 9.5x10^-3^ to 1.2x10^-3^ substitutions/site/year. This is similar to the value described in the cloning study [16], and to the rates found in other RNA viruses, such as HCV, with 1.44–1.92×10^−3^ substitutions/site/year [24].

It could be argued that these values may underestimate the true mutation rate because only the coding part of the genome was included. However, the HDAg encoding region, together with its self-complementary region, would represent most of the HDV genome, and it is likely that the 2 regions would evolve in a similar fashion to maintain the essential secondary structure of HDV RNA. Hence, we believe that the evolution rate reported in the analyzed region would be a good estimate of evolution in the complete genome. Nevertheless, this possibility should be confirmed by HDV quasispecies analysis based on complete genome sequencing, which should be possible using the upcoming “next-next” generation sequencing technology.

The high evolution rate in HDV RNA has been explained by increases in the RNA-Pol II mutation rate when acting on HDV RNA genomes [25]; nonetheless, the study provided no clear explanation of the experimental proofs undertaken to support this theory. In the present study, evolution was also evaluated for the genomes encoding S-HDAg and L-HDAg. Unedited genomes (encoding S-HDAg) had significantly higher evolution rates than edited genomes (paired-samples analysis, p = 0.002). This observation could indicate that unedited forms have higher fitness capacity than edited ones and may be in accordance with the replicative capacity of unedited genomes [26].

Interestingly, evolution rates in the HDV region studied showed a significant exponential decay with the years elapsed between samples. This suggests that in untreated chronic infection, the HDAg ORF initially adapts to the host by numerous changes in the quasispecies and after this initial adaptation, reaches a certain “steady state” of accumulated mutations. Decreases in the evolution rate over time have been described in cell culture systems [27,28], and the evolution rates in foot-and-mouth virus have been found to be higher in samples taken at close time points than at distant times [29]. To our knowledge, this is the first report of an exponential decay pattern in the evolution of human viral infection.

The present study attempted to identify viral factors associated with the evolution pattern in the region studied. The only significant finding was the number of transitions accumulated in the region. The other viral factors analyzed (quasispecies complexity and percentage of unedited and edited genomes) showed no relationship with evolution. As would be expected, transitions were the most common changes in the HDV region, and our results indicate that transitions may be responsible for higher evolution. Of the 4 possible transition combinations, U to C and C to U changes were the most common in the region studied, although they did not correlate with the evolution rate. U to C and C to U can be attributed to the ADAR1 or APOBEC enzymes (ADAR1 for A to G in the antigenome and APOBEC3 for C to U in the genome), reported to be enhanced in CHD mouse models [30]. These enzymes have neighbor preferences at 5’ and 3’ of the change [31–33]. We performed an analysis to define the type of nucleotide present at 5’ and at 3’ of the U to C and C to U changes in our sequences, but no clear pattern was observed (data not shown). Furthermore, although it cannot be excluded that ADAR1 or APOBEC can act under different situations in HDV than the one described, our results did not show a direct relationship of ADAR1 or APOBEC in terms of nucleotide neighbor preferences in the HDV region analyzed.

The 3 long-term studies showed that the percentage of unedited (amber codon) and edited (W codon) genomes fluctuated over the years. The presence of both types of particles with a predominance of those carrying unedited over edited genomes in our patient samples concurs with the chronic state of the infection and suggests that HDV may be a kind of dual infection (unedited plus edited HDV particles). The novelty of our study is that these 2 genomes were quantified. The values did not correlate with the evolution rate, possibly indicating that a balanced proportion is useful for the HDV quasispecies to maintain chronic infection. The results from this study led us to speculate that the presence of HBV and unedited and edited HDV particles could occur more easily in HDV superinfection (because HBV is already present) than in coinfection (which requires new entrance of the 3 different particles). However, this hypothesis would have to be confirmed in future studies analyzing patients with coinfection and superinfection.

The indices of quasispecies complexity were designed to characterize populations in ecology [34], but have also proven useful for characterizing HCV [35–37] and HBV in infection [14,38–40]. Evaluation of viral quasispecies complexity *per se* is of value to gain a greater understanding of viral infection. Because HDV is an RNA virus, it might be assumed that the complexity of the quasispecies would be similar to that of other RNA viruses, such as HCV. Nonetheless, the mode of HDV genome replication (RNA pol-II) and editing (ADAR1) by host enzymes, with lower error rates than with viral polymerases, suggests that HDV complexity might be lower than that of viruses replicating with their own polymerases without proofreading activity. However, the 6 indices selected to describe quasispecies complexity according to incidence, abundance, and functionality show that HDV complexity is similar to or higher than that of other RNA viruses [17,41,42] despite its particular mode of replication. In addition, we found that HDV quasispecies complexity was significantly lower when the amber/W codon was excluded from the analysis, suggesting that this codon may represent an artifact in the assessment of the HDV complexity. Nonetheless, no common pattern of quasispecies complexity was detected along longitudinal follow-up of the 3 cases analyzed. Quasispecies complexity evaluated separately in unedited and edited genomes showed no significant differences, although the fluctuating patterns were similar (Fig 5, panel B); hence, ADAR1 activity does not seem to result in differential variability between the genome types.

The indices of viral quasispecies complexity did not correlate with the evolution rate, although complexity presented a dynamic fluctuation (with peaks of increase and decrease). HDV seemed to continuously adapt to the host, but once a “steady state” of mutations had been achieved, the nucleotide substitutions generated did not accumulate. Hence, viral evolution must be considered a dynamic process. Another step forward to understanding the pattern of HDV evolution would be to analyze other viral infections in long-term studies and to investigate the effect of treatment depending on the phase of evolution.

In conclusion, this is the first study evaluating a region of HDV in human samples using an NGS method. Our results show that the evolution rate in this specific HDV genomic region studied during CHD ranged from 9.5x10^-3^ to 1.2x10^-3^ substitutions/site/year with a decay over the time elapsed. Nucleotide transition changes were responsible for the higher evolution rates in the 3 sequential studies. HDV quasispecies complexity in the region analyzed changed over 11.5 years of study, but these changes were not related to the evolution pattern. The percentages of unedited and edited genomes also fluctuated during chronic infection, but showed no relationship with the evolution rate. These findings indicate that the evolution of the region of the HDAg ORF in chronic infection is dynamic and decays exponentially until reaching a steady state of mutations.

## Supporting Information

S1 Fig Exponential decay pattern between the evolution rate and the time elapsed between samples, individually for each patient. (PPTX)

S2 Fig Exponential decay pattern between the evolution rate and the time elapsed between samples, in unedited and edited genomes. (PPTX)

S3 Fig Multidimensional data analysis. The set of quasispecies diversity indices was centered and scaled in the sequential samples from each patient (A1, B1, and C1). In a principal components analysis (PCA), the first 2 principal components summarizing quasispecies complexity were plotted (A2, B2, and C2). The evolution of these first 2 principal components is shown in another plot where each data point is labeled as the time elapsed since the baseline sample (A3, B3, and C3). Finally the matrix of population genetic distances among samples of each patient was submitted to Multidimensional Scaling and the samples were represented on the two first components, and labeled by the elapsed time since baseline (A4, B4, and C4).
(PPTX)

S1 Protocol Supplementary materials and methods. Detailed description of amplification of the HDV regions, UDPS data treatment (quasispecies complexity, evolution rate, accumulation of mutations and multidimensional data analysis).
(DOCX)

S1 Table Biosample accession numbers of the NGS sequencing data from the samples analyzed in this study. (DOCX)

S2 Table Data obtained from next-generation sequencing of the 29 samples. Number of sequences obtained and filtered, number and proportion of sequences with amber codon or W at the editing codon, and indices of quasispecies complexity (mutation frequency and nucleotide diversity) for the 29 sequential samples from long-term follow-up of the 3 patients.
(DOCX)

S3 Table Number of nucleotide changes detected in the 29 sequential samples. The number of changes in comparison with the dominant haplotype sequence from the first sample of each patient was calculated for the complete region (a), the complete region without the editing codon (b), unedited genomes (c), and edited genomes (d).
(DOCX)
