# Supplementary figures and images for: Evidence of an Exponential Decay Pattern of the Hepatitis Delta Virus Evolution Rate and Fluctuations in Quasispecies Complexity in Long-Term Studies of Chronic Delta Infection
Source: PLoS One. 2016 Jun 30;11(6):e0158557. doi: 10.1371/journal.pone.0158557 (PMC4928832; doi:10.1371/journal.pone.0158557)

## Slide 1
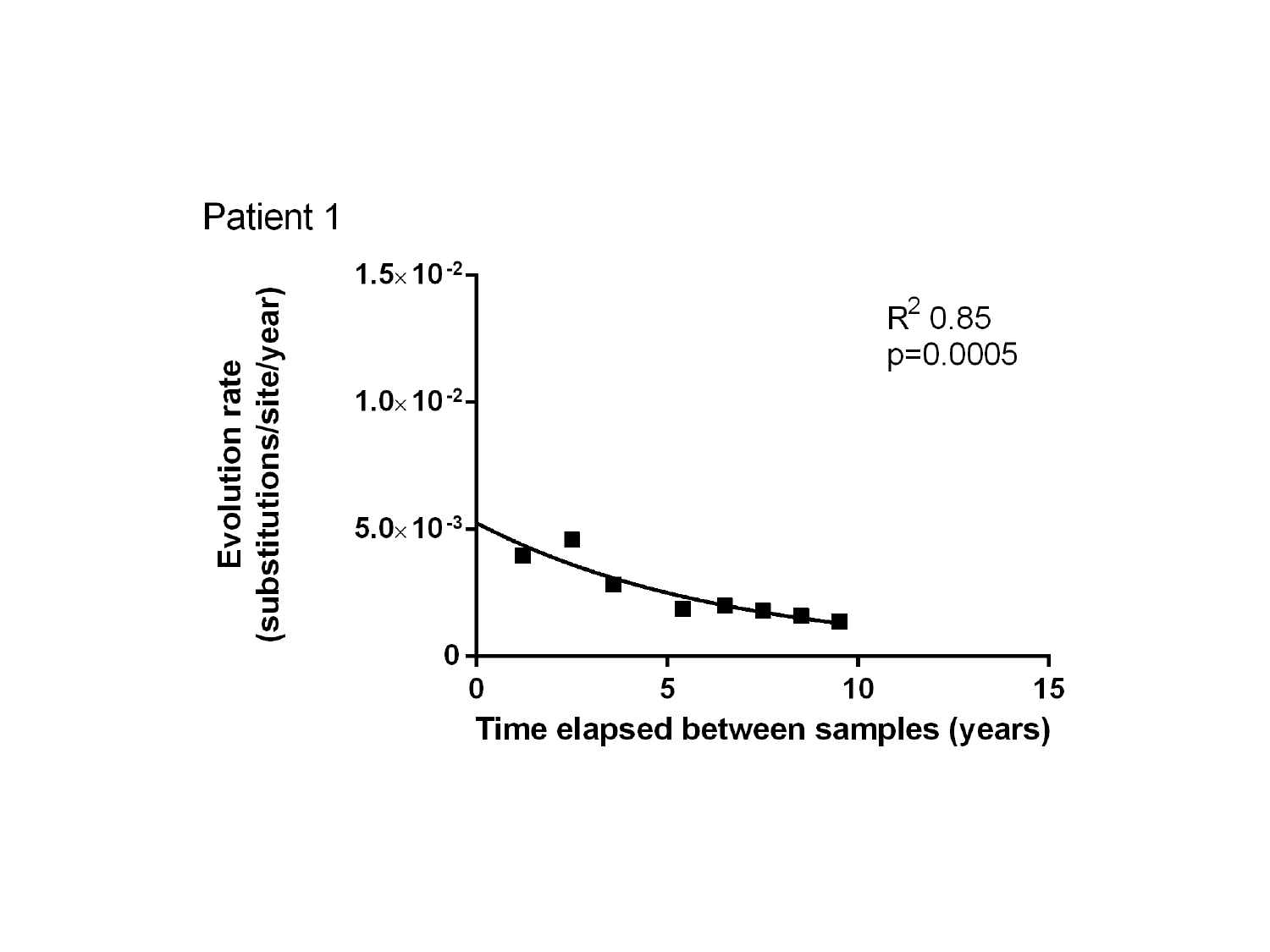

## Slide 2
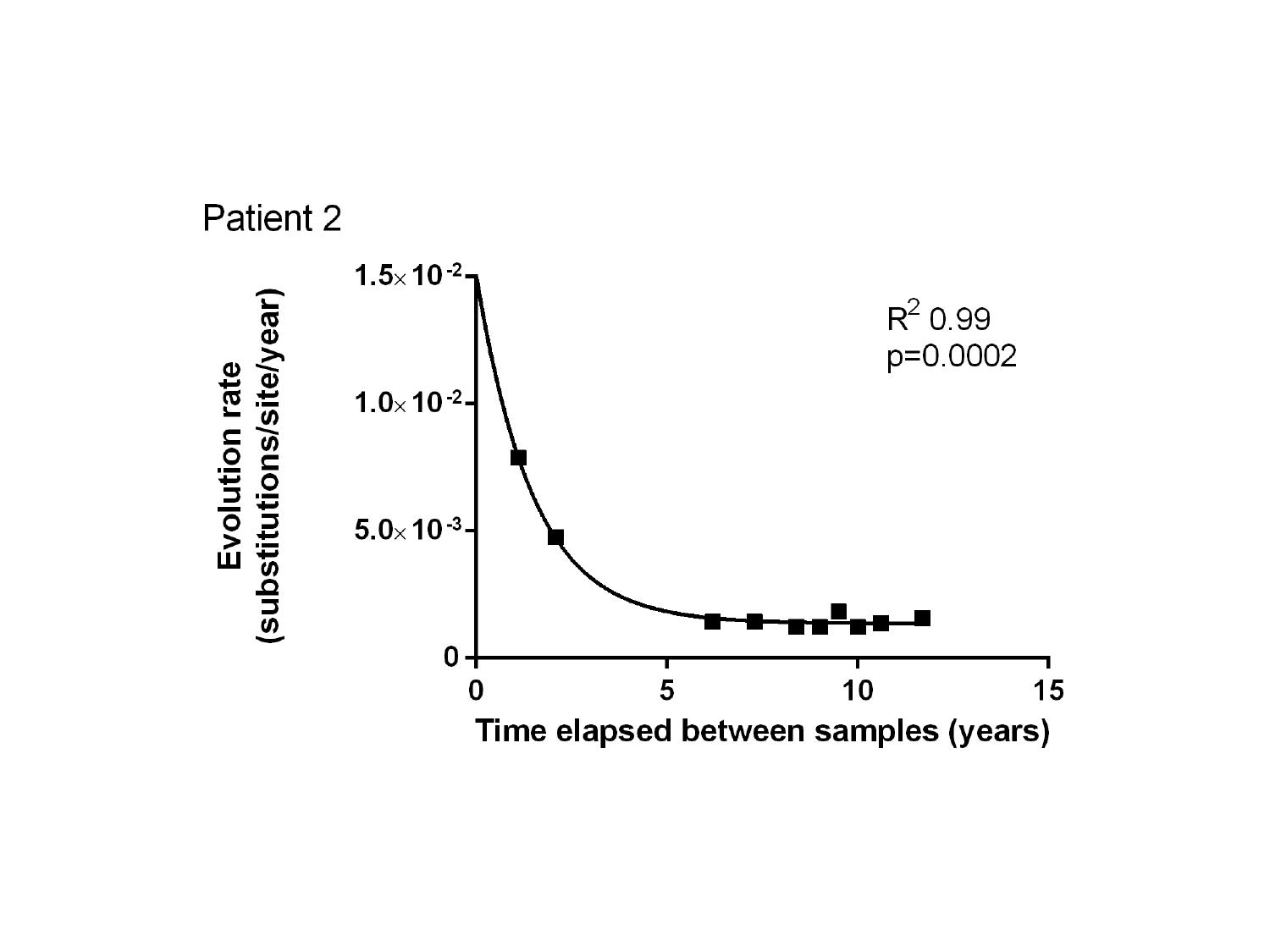

## Slide 3
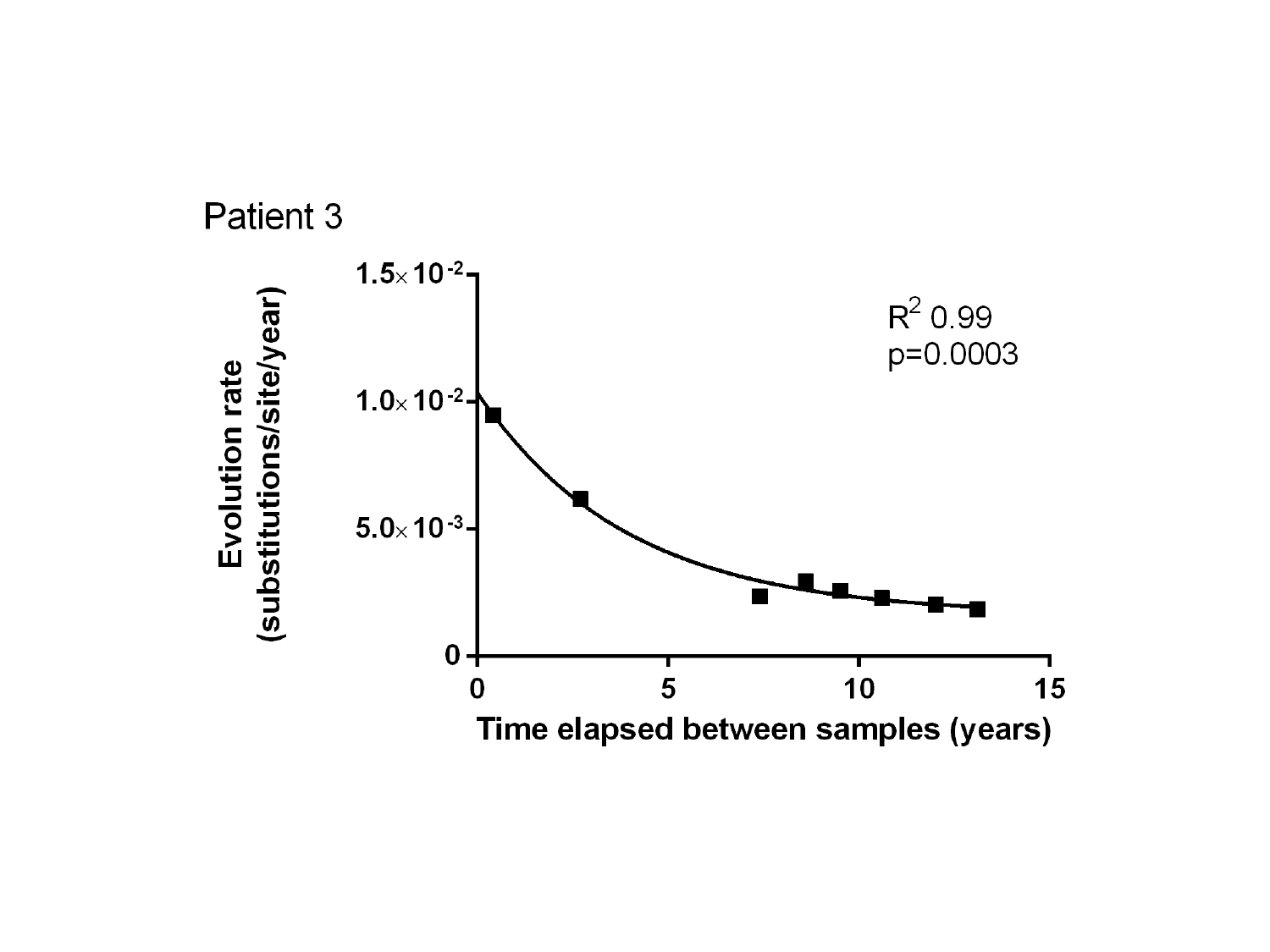

Supplement: S1 Fig — (PPTX) [file pone.0158557.s001.pptx]

## Slide 1
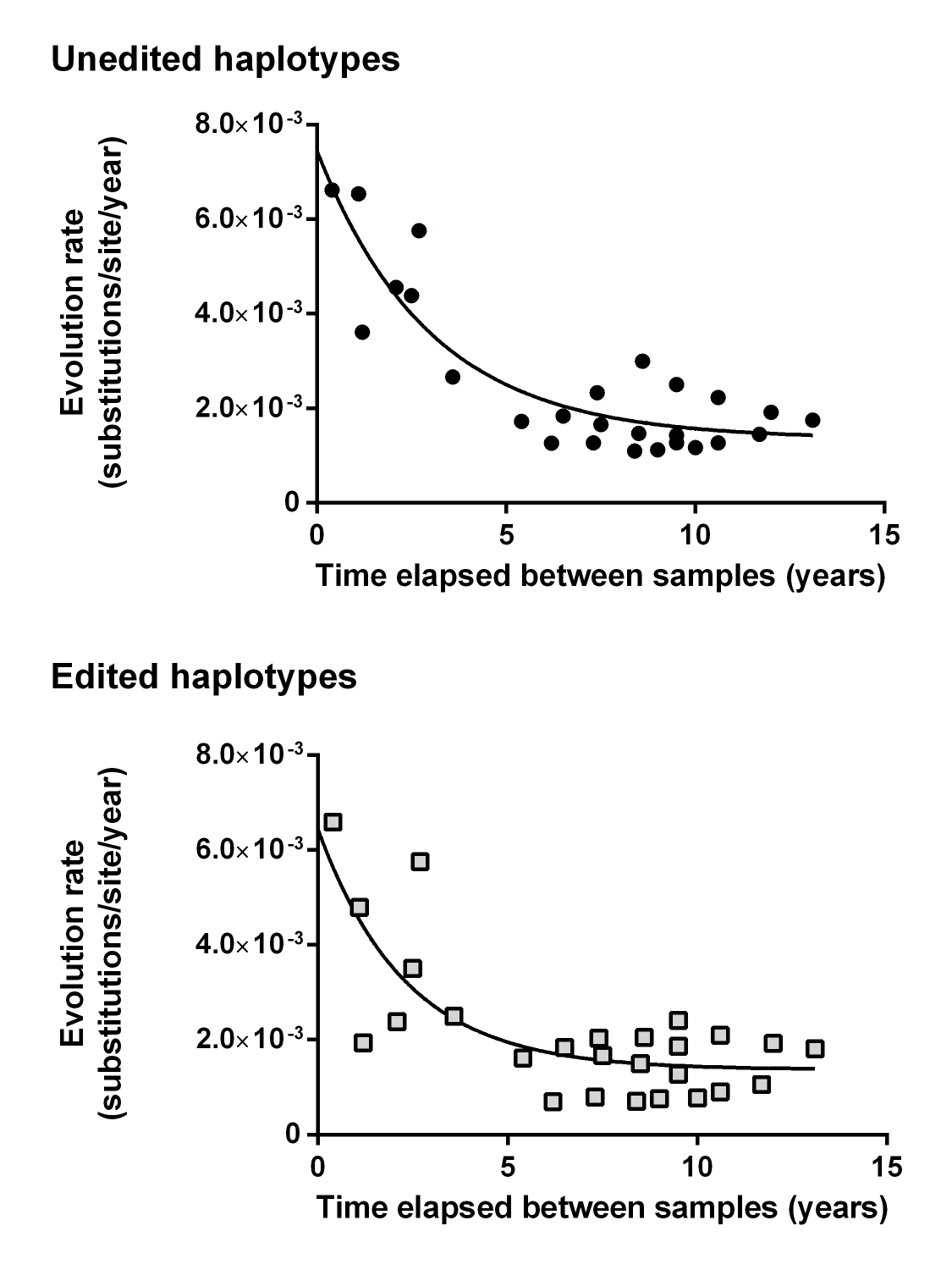

Supplement: S2 Fig — (PPTX) [file pone.0158557.s002.pptx]

## Slide 1
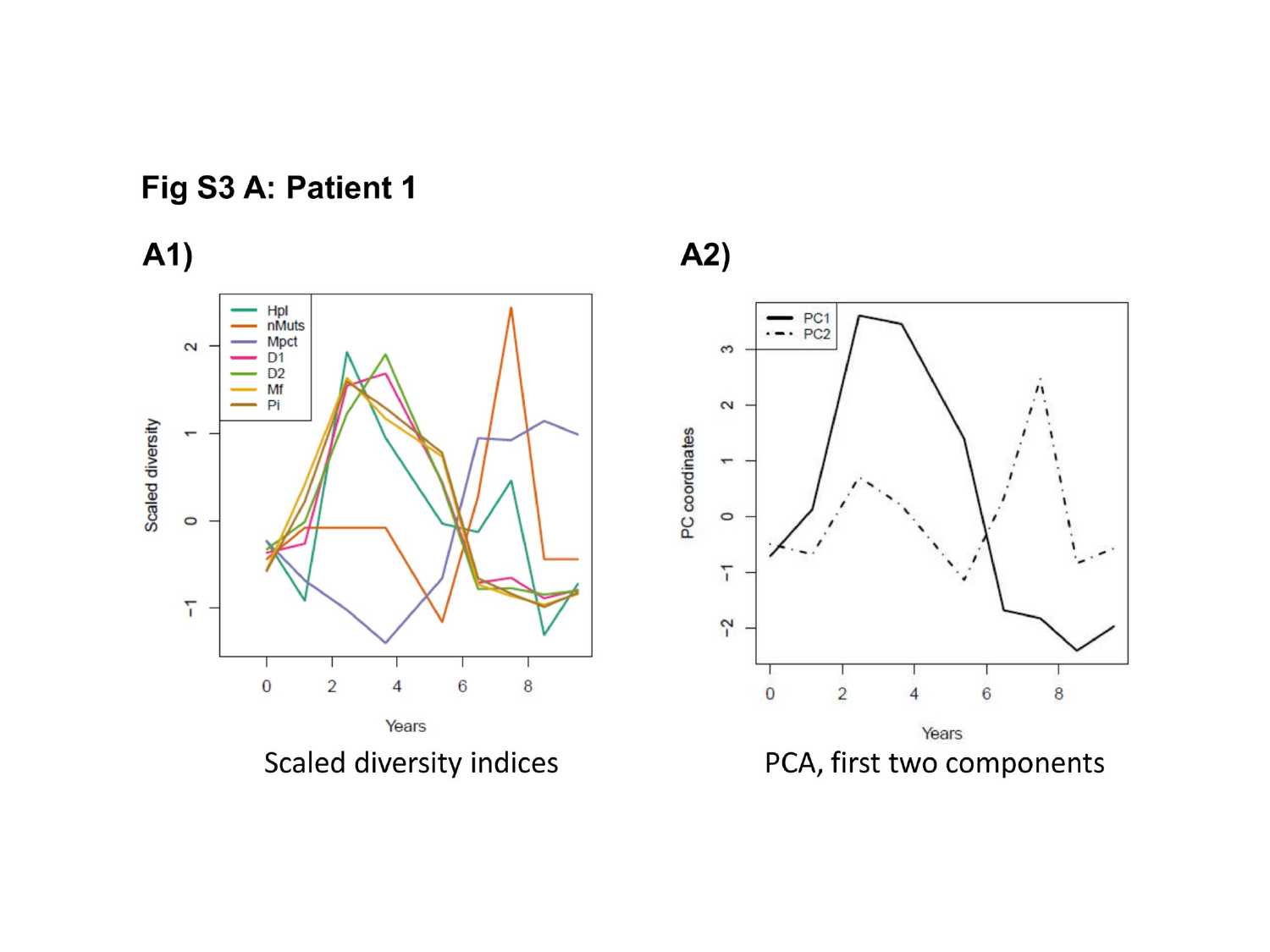

## Slide 2
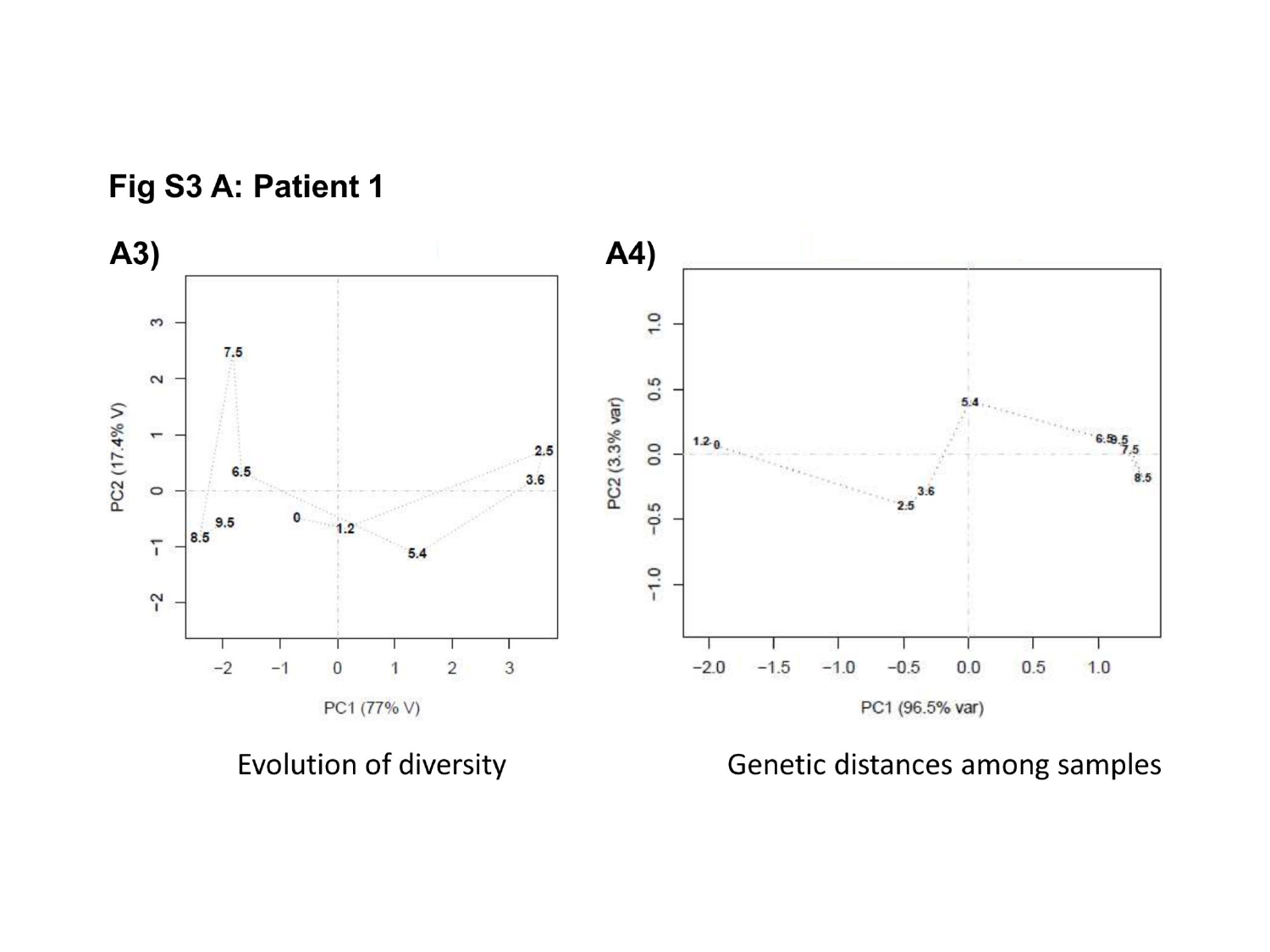

## Slide 3
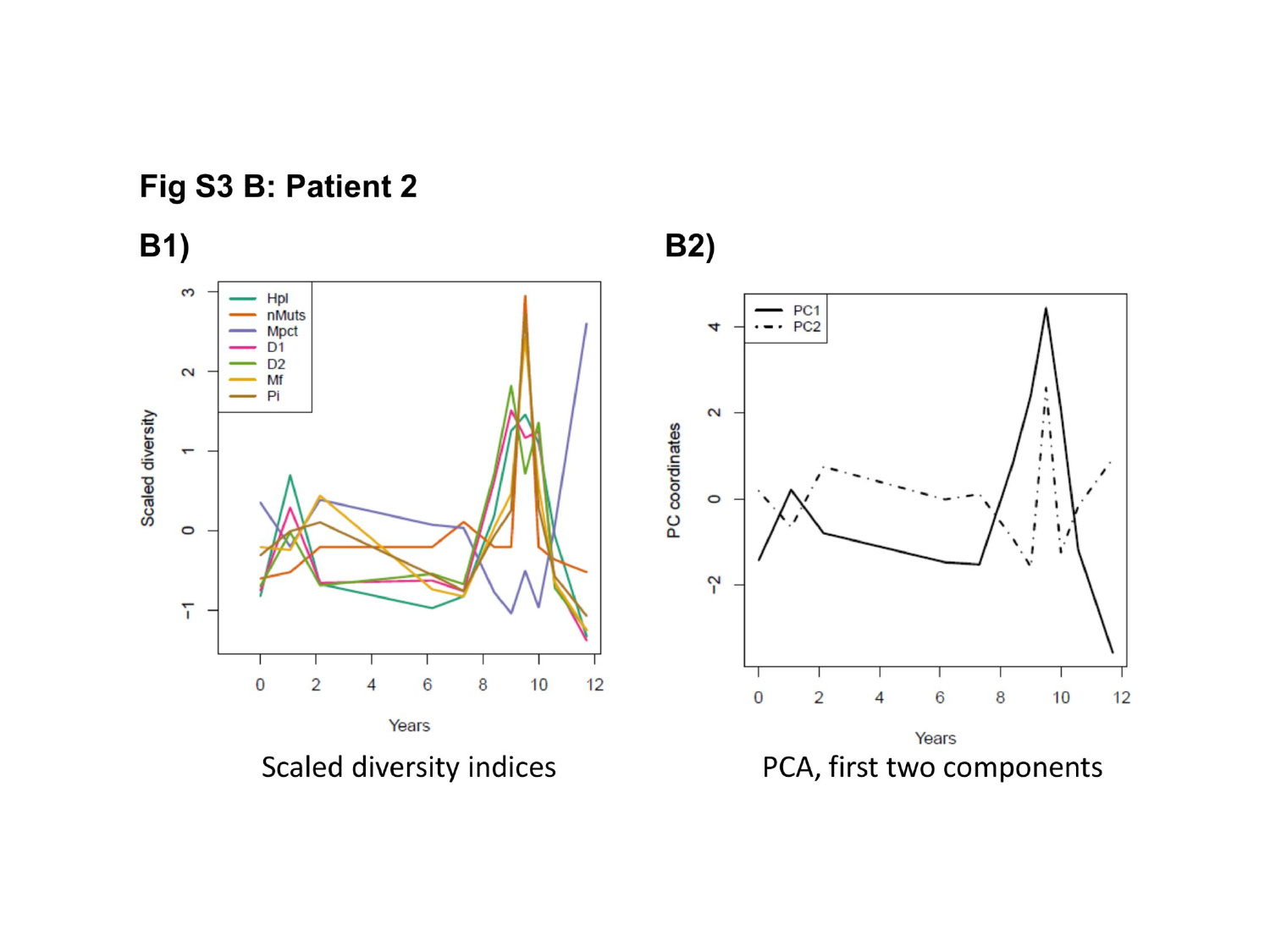

## Slide 4
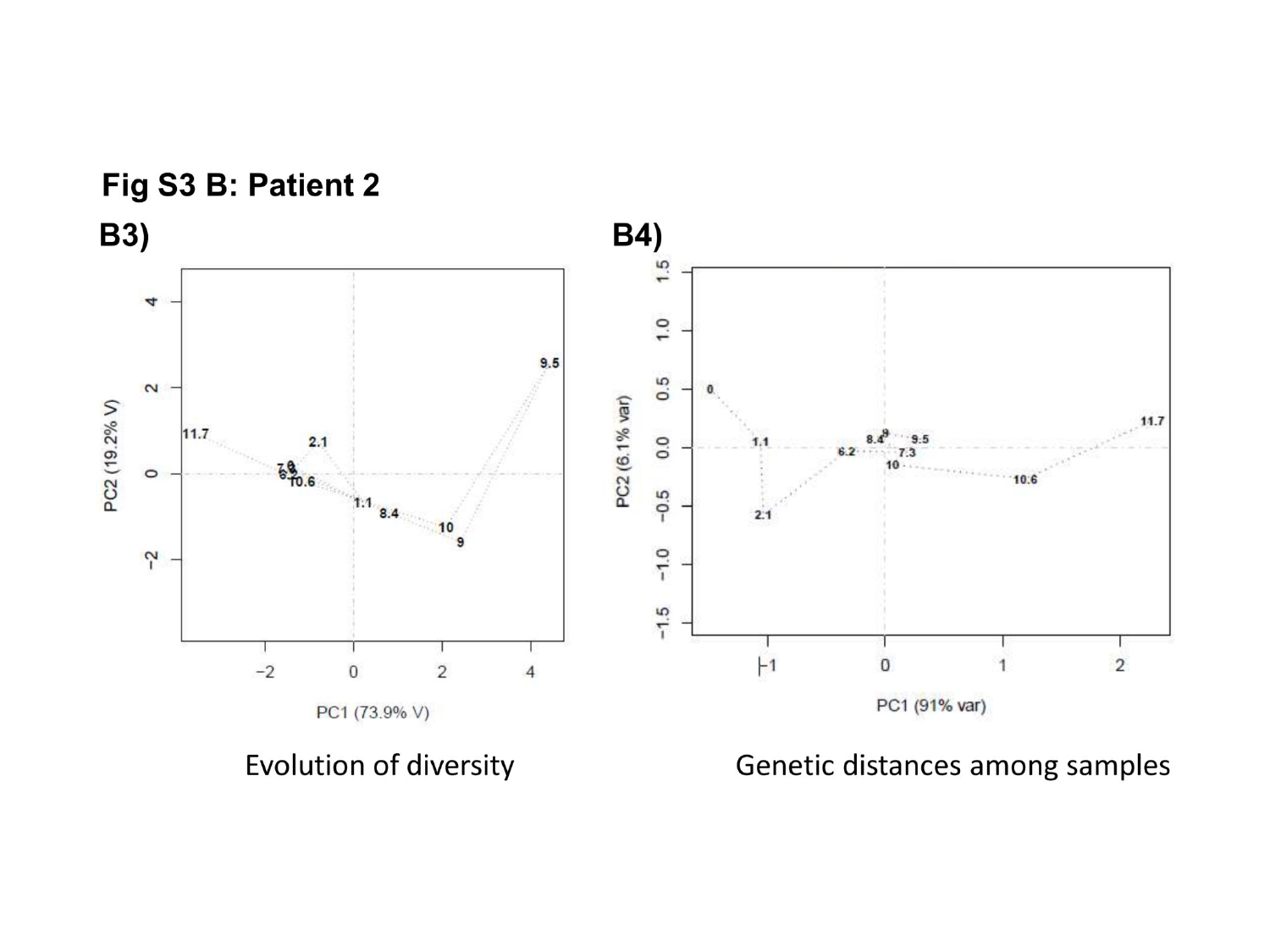

## Slide 5
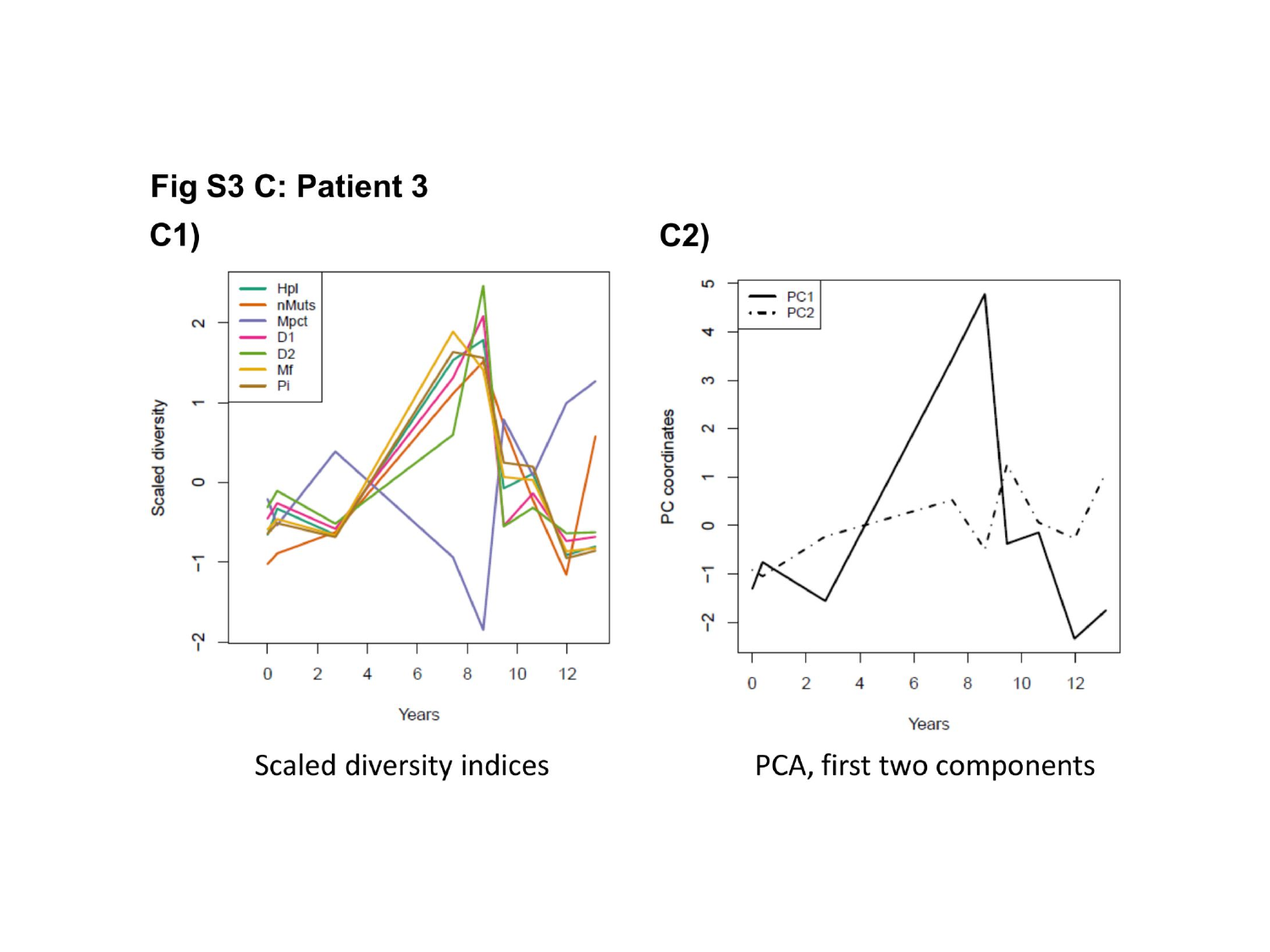

## Slide 6
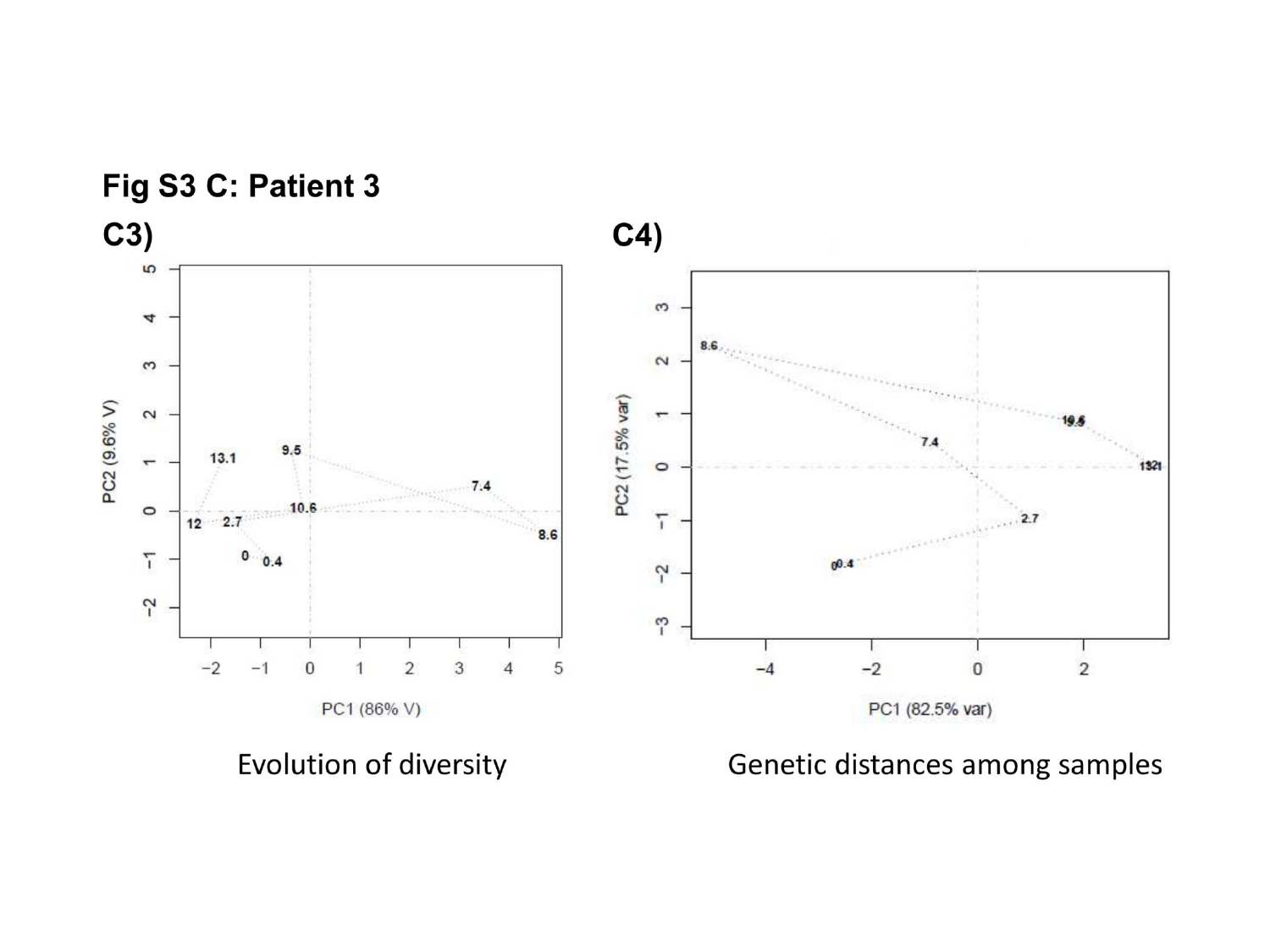

Supplement: S3 Fig — The set of quasispecies diversity indices was centered and scaled in the sequential samples from each patient (A1, B1, and C1). In a principal components analysis (PCA), the first 2 principal components summarizing quasispecies complexity were plotted (A2, B2, and C2). The evolution of these first 2 principal components is shown in another plot where each data point is labeled as the time elapsed since the baseline sample (A3, B3, and C3). Finally the matrix of population genetic distances among samples of each patient was submitted to Multidimensional Scaling and the samples were represented on the two first components, and labeled by the elapsed time since baseline (A4, B4, and C4). (PPTX) [file pone.0158557.s003.pptx]
