# Supplementary material for: Evidence of an Exponential Decay Pattern of the Hepatitis Delta Virus Evolution Rate and Fluctuations in Quasispecies Complexity in Long-Term Studies of Chronic Delta Infection
Source: PLoS One. 2016 Jun 30;11(6):e0158557. doi: 10.1371/journal.pone.0158557 (PMC4928832; doi:10.1371/journal.pone.0158557)
Supplement: S1 Protocol — Detailed description of amplification of the HDV regions, UDPS data treatment (quasispecies complexity, evolution rate, accumulation of mutations and multidimensional data analysis). (DOCX) [file pone.0158557.s004.docx]

**Supplementary Materials and Methods**

**Amplification of the HDV region**

HDV RNA was extracted from serum samples with a total nucleic acid isolation kit (TNAI, Roche Diagnostics, GmbH, Mannheim, Germany). Ten μL of the extracted HDV RNA was denatured in the presence of 10 mM of primer 771 (771-reverse, 5’ CGGTCCCCTCGGAATGTTG). The denatured HDV RNA with the primer was then reverse transcribed using Accuscript HiFi reverse transcriptase (Agilent Technologies, Santa Clara, USA) according to manufacturer’s instructions.

cDNA was amplified in a first PCR reaction, using 0.2 µM of the forward and reverse primer (891-forward, 5’ AGGTCGGACCGCGAGGAGGT and 339- reverse, 5’ GCTGAAGGGGTCCTCTGGAGGTG), 1x Pfu Ultra II polymerase buffer, 0.25 mM of each dNTP, and 2.5 U of Pfu Ultra II DNA polymerase (Stratagene, Agilent Technologies) in a final volume of 50 µL. After a single denaturation step of 2 min at 95ºC, samples underwent 40 cycles of 20 s at 95ºC, 20 s at 54ºC, and 45 s at 72ºC, and a single final 3-min step at 72ºC.

The nested PCR was performed with primers that included an M13 universal adaptor sequence at the 5’ end (M13 912, 5’ *GTTGTAAAACGACGGCCAGT*GAGATGCCATGCCGACCCGAAGAG and M13 1298, 5’*CACAGGAAACAGCTATGACC*CGACGAAGGAAGGCCCTCGAGAAC). Briefly, PCR was performed using 1x Pfu Ultra II polymerase buffer, 0.25 mM of each dNTP, 0.2 µM of forward and reverse primer, and 2.5 U of Pfu Ultra II DNA polymerase (Stratagene, Agilent Technologies) in a final volume of 15 µL. After a single denaturation step of 2 min at 95ºC, samples underwent 35 cycles of 20 s at 95ºC, 20 s at 63ºC, and 15s at 72ºC, and a single final 3-min step at 72ºC.

An additional PCR was performed using a specific pair of primers for each sample: sense 5’-CGTATCGCCTCCCTCGCGCCATCAG-**MID**-***GTAAAACGACGGCCAGT***-3’ and anti-sense 5’-CTATGCGCCTTGCCAGCCCGCTCAG-**MID**-***AACAGCTATGACCATG****-*3’. These primers contain the 5’ sequences A and B, which are adaptors for the elements of the UDPS system, followed by a unique identifier that enables grouping of the sequences derived from each sample (multiplex identifier sequences [MID], in bold), and the same M13 universal adaptor sequences as the primers used in the first PCR (in bold and italics). To avoid errors and facilitate the complex process of using different primer pairs for each sample, universal nested primers were lyophilized and preloaded in 96-well PCR plates.

The PCR products were purified with AMPure beads (Beckman Coulter, USA). The quality of the DNA was verified with the Bioanalyzer (Agilent DNA 1000, Life Technologies, Oregon, USA) and was quantified using the Quan-iT Picogreen dsDNA Assay kit (Life Technologies, Oregon, USA). Deep sequencing was performed with the 454/GS-Junior platform (Roche, Branford, CT 06405, USA), using titanium chemistry (GS-Junior Titanium Sequencing Kit).

**UDPS data treatment**

1) Raw data: The fasta files provided by the 454 Junior were used as raw data.

2) Demultiplexing: Identification of the MID sequence, M13 primer, and amplicon-specific primers was used to split the reads into different fasta files. One mismatch was tolerated in the MID identification and three mismatches in the specific primer identification.

3) General quality filter: Reads that did not cover the full amplicon, and those that showed more than 2 indeterminations or more than 3 gaps with respect the dominant haplotype were excluded. The remaining indeterminations and gaps were repaired as per the dominant haplotype in each sample.

4) Forward and reverse intersection: Only haplotypes common to the forward and reverse strand present in abundances of 0.1% and higher were accepted. The final frequency for each haplotype is the sum of the reads observed in each strand.

5) Final abundance filter: haplotypes with abundances below 0.25% were excluded.

6) Translation to amino acid sequences: Haplotypes resulting from step 5 were translated into amino acid sequences.

7) Identification of amino acid variants: The multiple alignment of the amino acid sequences obtained in the previous step was used to find and quantify the variants as point mutations with corresponding abundances.

8) Diversity analysis: The nucleotide haplotypes resulting from step 4 were used for diversity quantification and analysis. Down-sampling followed by fringe-trimming [1] was used with each group of samples to make their diversity indices comparable. Down-sampling was done by scaling to the smallest coverage of any sample in the group.

All these steps were performed with in-house developed scripts in R [1–4].

**Quasispecies diversity indices**

The complexity of the mutant spectrum of each quasispecies was characterized through the use of two incidence-based indices, number of haplotypes and number of mutations; two abundance-based indices, Hill numbers of order 1 and 2, corresponding to a transformation of the Shannon entropy index and Simpson index; and two functional indices, mutation frequency and nucleotide diversity.

The number of haplotypes is the number of unique sequences in the set of all reads. The number of mutations is the number of different mutations that are observed in the multiple sequence alignment of all haplotypes, with respect to the dominant or master haplotype. Hill numbers are a generalization of abundance-based diversity indices with an exponent of order q, which influences the sensitivity of the diversity index with respect to the rare haplotypes in the quasispecies. These numbers are favored by exhibiting the doubling property, being less prone to saturability, and by having common units with intuitive meaning: number of equally abundant haplotypes which show the same diversity measure as the inspected sample.

Mutation frequency is the fraction of nucleotides in the quasispecies that are different with respect to the most abundant haplotype in the sample. Nucleotide diversity is expressed as the mean fraction of different nucleotides between all pairs of molecules in the sample.

**Evolution rate**

The dominant haplotype of the baseline sample for each patient was taken as reference, and the evolution at each time point in the longitudinal study was computed as the frequency of substitutions in each quasispecies with respect to the corresponding baseline reference. The abundance of each haplotype in the computation was considered a surrogate of its fitness and the ability to fix the new mutations within the quasispecies.

**Accumulation of mutations**

This represents the dynamics in the number of mutations observed in the quasispecies regardless of their abundance, but taking into account all observed haplotypes, and with respect to the dominant haplotype of the baseline sample.

**Multidimensional data analysis**

The set of diversity indices was centered and scaled to a standard deviation of 1, so that all indices appeared on the same scale. The centered and scaled indices for the sequential samples of each patient were plotted to show the changes occurring in quasispecies complexity (Fig. S3 A1, B1 and C1). In addition, in a principal components analysis (PCA) [5] of the scaled matrix for each patient, the first two principal components were plotted to summarize the quasispecies complexity on two axes showing the highest variation (Fig. S3 A2, B2 and C2). The evolution of these first two principal components is shown in a second plot where each data point is labeled as the time elapsed since the baseline sample (Fig. S3 A3, B3 and C3).

Furthermore, the population genetic distances [6] between each pair of samples were computed for all three patients and represented on a plot of the two first components in a multidimensional scaling [5], where the genetic distance between each pair of samples is visualized (Fig S3 A4, B4 and C4). In this plot the samples are also labeled as the time elapsed since the baseline sample.

**References:**

1. Gregori J, Salicrú M, Domingo E, Sanchez A, Esteban JI, Rodríguez-Frías F, et al. Inference with viral quasispecies diversity indices: Clonal and NGS approaches. Bioinformatics. 2014;30: 1104–1111. doi:10.1093/bioinformatics/btt768

2. Ramírez C, Gregori J, Buti M, Tabernero D, Camós S, Casillas R, et al. A comparative study of ultra-deep pyrosequencing and cloning to quantitatively analyze the viral quasispecies using hepatitis B virus infection as a model. Antiviral Res. 2013;98: 273–83. doi:10.1016/j.antiviral.2013.03.007

3. Homs M, Caballero A, Gregori J, Tabernero D, Quer J, Nieto L, et al. Clinical Application of Estimating Hepatitis B Virus Quasispecies Complexity by Massive Sequencing: Correlation between Natural Evolution and On-Treatment Evolution. PLoS One. 2014;9: e112306. doi:10.1371/journal.pone.0112306

4. Gregori J, Esteban JI, Cubero M, Garcia-Cehic D, Perales C, Casillas R, et al. Ultra-deep pyrosequencing (UDPS) data treatment to study amplicon HCV minor variants. PLoS One. 2013;8: e83361. doi:10.1371/journal.pone.0083361

5. Rencher A. Methods of Multivariate Analysis. 2nd ed. New York: John Wiley & Sons; 2002.

6. Nei M. Molecular Evolutionary Genetics. New York: Columbia University Press; 1987.
