# Supplementary material for: Evidence of an Exponential Decay Pattern of the Hepatitis Delta Virus Evolution Rate and Fluctuations in Quasispecies Complexity in Long-Term Studies of Chronic Delta Infection
Source: PLoS One. 2016 Jun 30;11(6):e0158557. doi: 10.1371/journal.pone.0158557 (PMC4928832; doi:10.1371/journal.pone.0158557)
Supplement: S1 Table — (DOCX) [file pone.0158557.s005.docx]

| **Patient** | **Sample ID** | **Sample ID Biosample** | **Biosample accession number** |
| --- | --- | --- | --- |
| 1 | 1 | P01S01 | SAMN04958056 |
| 1 | 2 | P01S02 | SAMN04958057 |
| 1 | 3 | P01S03 | SAMN04958058 |
| 1 | 4 | P01S04 | SAMN04958059 |
| 1 | 5 | P01S05 | SAMN04958060 |
| 1 | 6 | P01S06 | SAMN04958061 |
| 1 | 7 | P01S07 | SAMN04958062 |
| 1 | 8 | P01S08 | SAMN04958063 |
| 1 | 9 | P01S09 | SAMN04958064 |
| 2 | 1 | P02S01 | SAMN04958065 |
| 2 | 2 | P02S02 | SAMN04958066 |
| 2 | 3 | P02S03 | SAMN04958067 |
| 2 | 4 | P02S04 | SAMN04958068 |
| 2 | 5 | P02S05 | SAMN04958069 |
| 2 | 6 | P02S06 | SAMN04958070 |
| 2 | 7 | P02S07 | SAMN04958071 |
| 2 | 8 | P02S08 | SAMN04958072 |
| 2 | 9 | P02S09 | SAMN04958073 |
| 2 | 10 | P02S10 | SAMN04958074 |
| 2 | 11 | P02S11 | SAMN04958075 |
| 3 | 1 | P03S01 | SAMN04958076 |
| 3 | 2 | P03S02 | SAMN04958077 |
| 3 | 3 | P03S03 | SAMN04958078 |
| 3 | 4 | P03S04 | SAMN04958079 |
| 3 | 5 | P03S05 | SAMN04958080 |
| 3 | 6 | P03S06 | SAMN04958081 |
| 3 | 7 | P03S07 | SAMN04958082 |
| 3 | 8 | P03S08 | SAMN04958083 |
| 3 | 9 | P03S09 | SAMN04958084 |

**S1 Table** BioSample accession numbers of the NGS sequencing data of the samples analyzed in this study. Sample identifiers provided in the paper (Sample ID) and unique identifiers for each Biosample (Sample ID Biosample) are also shown.
