# Supplementary material for: Evidence of an Exponential Decay Pattern of the Hepatitis Delta Virus Evolution Rate and Fluctuations in Quasispecies Complexity in Long-Term Studies of Chronic Delta Infection
Source: PLoS One. 2016 Jun 30;11(6):e0158557. doi: 10.1371/journal.pone.0158557 (PMC4928832; doi:10.1371/journal.pone.0158557)
Supplement: S2 Table — Number of sequences obtained and filtered, number and proportion of sequences with amber codon or W at the editing codon, and indices of quasispecies complexity (mutation frequency and nucleotide diversity) for the 29 sequential samples from long-term follow-up of the 3 patients. (DOCX) [file pone.0158557.s006.docx]

**S2 Table** Number of sequences obtained and number of sequences filtered for analysis in the 29 sequential samples obtained over follow-up in the 3 patients. The type of codon at the amber/W codon, their number and percentage, and the parameters of HDV quasispecies complexity (mutation frequency and nucleotide diversity) are also presented

| **Patient** | **Sample ID** | **Collection date** | **NGS Raw data** | **Filtered reads** | **Codons at the editing codon** | **Number of sequences** | **Percentage of genome** | **Mutation frequency (substitutions/site)** | **Nucleotide diversity (substitutions/site)** |
| --- | --- | --- | --- | --- | --- | --- | --- | --- | --- |
| **1** | 1 | 01/08/2005 | 6972 | 3725 | Stop | 2505 | 67.2 | 2.23E-03 | 3.60E-03 |
|  |  |  |  |  | Tryptophan | 1220 | 32.8 | 1.98E-03 | 3.11E-03 |
|  | 2 | 03/10/2006 | 5230 | 3274 | Stop | 2130 | 65.1 | 4.28E-03 | 6.27E-03 |
|  |  |  |  |  | Tryptophan | 1144 | 34.9 | 2.27E-03 | 3.46E-03 |
|  | 3 | 17/01/2008 | 6100 | 3319 | Stop | 1850 | 55.7 | 6.21E-03 | 8.84E-03 |
|  |  |  |  |  | Tryptophan | 1469 | 44.3 | 8.63E-03 | 9.43E-03 |
|  | 4 | 23/03/2009 | 2734 | 1216 | Stop | 924 | 76.0 | 6.75E-03 | 9.01E-03 |
|  |  |  |  |  | Tryptophan | 292 | 24.0 | 8.47E-03 | 8.73E-03 |
|  | 5 | 16/12/2010 | 5729 | 2578 | Stop | 1752 | 68.0 | 5.27E-03 | 7.16E-03 |
|  |  |  |  |  | Tryptophan | 826 | 32.0 | 5.61E-03 | 7.21E-03 |
|  | 6 | 20/01/2012 | 6247 | 3387 | Stop | 2321 | 68.5 | 1.79E-03 | 3.27E-03 |
|  |  |  |  |  | Tryptophan | 1066 | 31.5 | 1.74E-03 | 3.16E-03 |
|  | 7 | 24/01/2013 | 3968 | 2150 | Stop | 1490 | 69.3 | 1.48E-03 | 2.79E-03 |
|  |  |  |  |  | Tryptophan | 660 | 30.7 | 1.21E-03 | 2.31E-03 |
|  | 8 | 28/01/2014 | 2390 | 1209 | Stop | 864 | 71.5 | 1.38E-03 | 2.55E-03 |
|  |  |  |  |  | Tryptophan | 345 | 28.5 | 8.08E-04 | 1.55E-03 |
|  | 9 | 09/02/2015 | 8944 | 4988 | Stop | 3391 | 68.0 | 1.56E-03 | 2.88E-03 |
|  |  |  |  |  | Tryptophan | 1597 | 32.0 | 1.43E-03 | 2.67E-03 |

| **Patient** | **Sample ID** | **Collection date** | **NGS Raw data** | **Filtered reads** | **Codons at the editing codon** | **Number of sequences** | **Percentage of genome** | **Mutation frequency (substitutions/site)** | **Nucleotide diversity (substitutions/site)** |
| --- | --- | --- | --- | --- | --- | --- | --- | --- | --- |
| **2** | 1 | 16/09/1999 | 7594 | 4686 | Stop | 3467 | 74.0 | 3.18E-03 | 4.48E-03 |
|  |  |  |  |  | Tryptophan | 1219 | 26.0 | 4.46E-03 | 5.24E-03 |
|  | 2 | 11/10/2000 | 14529 | 8909 | Stop | 6090 | 68.4 | 3.79E-03 | 6.21E-03 |
|  |  |  |  |  | Tryptophan | 2819 | 31.6 | 5.13E-03 | 8.00E-03 |
|  | 3 | 07/11/2001 | 4284 | 2206 | Stop | 1637 | 74.2 | 4.65E-03 | 6.11E-03 |
|  |  |  |  |  | Tryptophan | 569 | 25.8 | 6.16E-03 | 7.32E-03 |
|  | 4 | 15/11/2005 | 2456 | 1262 | Stop | 828 | 65.6 | 2.67E-03 | 4.50E-03 |
|  |  |  |  |  | Tryptophan | 434 | 34.4 | 2.65E-03 | 4.35E-03 |
|  | 5 | 02/01/2007 | 4086 | 1834 | Stop | 1108 | 60.4 | 2.27E-03 | 3.81E-03 |
|  |  |  |  |  | Tryptophan | 726 | 39.6 | 2.15E-03 | 3.54E-03 |
|  | 6 | 06/02/2008 | 4326 | 1545 | Stop | 1001 | 64.8 | 4.89E-03 | 6.60E-03 |
|  |  |  |  |  | Tryptophan | 544 | 35.2 | 4.34E-03 | 6.03E-03 |
|  | 7 | 16/09/2008 | 12700 | 6100 | Stop | 4249 | 69.7 | 6.05E-03 | 7.75E-03 |
|  |  |  |  |  | Tryptophan | 1851 | 30.3 | 6.06E-03 | 7.76E-03 |
|  | 8 | 18/03/2009 | 14569 | 5986 | Stop | 3863 | 64.5 | 8.15E-03 | 1.21E-02 |
|  |  |  |  |  | Tryptophan | 2123 | 35.5 | 1.78E-02 | 2.65E-02 |
|  | 9 | 10/09/2009 | 8484 | 4078 | Stop | 3192 | 78.3 | 6.73E-03 | 8.55E-03 |
|  |  |  |  |  | Tryptophan | 886 | 21.7 | 5.53E-03 | 7.42E-03 |
|  | 10 | 09/04/2010 | 19866 | 12239 | Stop | 6535 | 53.4 | 2.61E-03 | 4.51E-03 |
|  |  |  |  |  | Tryptophan | 5704 | 46.6 | 2.57E-03 | 4.39E-03 |
|  | 11 | 01/06/2011 | 11761 | 7764 | Stop | 1351 | 17.4 | 4.24E-03 | 5.94E-03 |
|  |  |  |  |  | Tryptophan | 6413 | 82.6 | 6.74E-04 | 1.28E-03 |

| **Patient** | **Sample ID** | **Collection date** | **NGS Raw data** | **Filtered reads** | **Codons at the editing codon** | **Number of sequences** | **Percentage of genome** | **Mutation frequency (substitutions/site)** | **Nucleotide diversity (substitutions/site)** |
| --- | --- | --- | --- | --- | --- | --- | --- | --- | --- |
| **3** | 1 | 24/10/2001 | 7391 | 3874 | Stop | 2480 | 64.0 | 1.98E-03 | 3.21E-03 |
|  |  |  |  |  | Tryptophan | 1394 | 36.8 | 1.61E-03 | 2.60E-03 |
|  | 2 | 12/03/2002 | 7316 | 3827 | Stop | 2523 | 65.9 | 2.53E-03 | 3.92E-03 |
|  |  |  |  |  | Tryptophan | 1304 | 34.1 | 2.51E-03 | 3.76E-03 |
|  | 3 | 09/07/2004 | 10136 | 5440 | Stop | 3317 | 61.0 | 1.56E-03 | 2.89E-03 |
|  |  |  |  |  | Tryptophan | 2123 | 39.0 | 1.28E-03 | 2.38E-03 |
|  | 4 | 26/03/2009 | 12985 | 2692 | Stop | 2105 | 78.2 | 1.58E-02 | 1.99E-02 |
|  |  |  |  |  | Tryptophan | 587 | 21.8 | 4.49E-03 | 7.72E-03 |
|  | 5 | 11/06/2010 | 14090 | 3026 | Stop | 2772 | 91.6 | 1.08E-02 | 1.66E-02 |
|  |  |  |  |  | Tryptophan | 254 | 8.4 | 1.63E-02 | 1.74E-02 |
|  | 6 | 07/04/2011 | 11049 | 5098 | Stop | 3505 | 68.8 | 6.83E-03 | 1.19E-02 |
|  |  |  |  |  | Tryptophan | 1593 | 31.2 | 1.56E-03 | 2.99E-03 |
|  | 7 | 07/06/2012 | 4770 | 1409 | Stop | 981 | 69.6 | 5.50E-03 | 9.80E-03 |
|  |  |  |  |  | Tryptophan | 428 | 30.4 | 3.18E-03 | 5.83E-03 |
|  | 8 | 08/10/2013 | 12224 | 7607 | Stop | 4568 | 60.0 | 5.24E-04 | 1.02E-03 |
|  |  |  |  |  | Tryptophan | 3039 | 40.0 | 5.83E-04 | 1.14E-03 |
|  | 9 | 09/12/2014 | 9151 | 5688 | Stop | 3724 | 65.5 | 7.43E-04 | 1.46E-03 |
|  |  |  |  |  | Tryptophan | 1964 | 34.5 | 1.41E-03 | 2.66E-03 |
