# Supplementary material for: Evidence of an Exponential Decay Pattern of the Hepatitis Delta Virus Evolution Rate and Fluctuations in Quasispecies Complexity in Long-Term Studies of Chronic Delta Infection
Source: PLoS One. 2016 Jun 30;11(6):e0158557. doi: 10.1371/journal.pone.0158557 (PMC4928832; doi:10.1371/journal.pone.0158557)
Supplement: S3 Table — The number of changes in comparison with the dominant haplotype sequence from the first sample of each patient was calculated for the complete region (a), the complete region without the editing codon (b), unedited genomes (c), and edited genomes (d). (DOCX) [file pone.0158557.s007.docx]

**S3 Table** Number nucleotide changes detected in the 29 sequential samples in comparison with the dominant haplotype sequence from the first sample of each patient. The number of changes was calculated for the complete region (a), complete region without the editing codon (b), unedited genomes (c), and edited genomes (d).

| **Pt** | **Sample** | 1. **Complete region (360-bp)** | | | | | | | | | | | | | | | |
| --- | --- | --- | --- | --- | --- | --- | --- | --- | --- | --- | --- | --- | --- | --- | --- | --- | --- |
|  |  | AA | AC | AG | AT | CA | CC | CG | CT | GA | GC | GG | GT | TA | TC | TG | TT |
| **1** | 1 | 2958 | 0 | 32 | 13 | 0 | 3237 | 0 | 0 | 34 | 0 | 4295 | 0 | 0 | 0 | 0 | 1599 |
|  | 2 | 2273 | 1 | 25 | 11 | 1 | 2486 | 2 | 1 | 44 | 2 | 3283 | 1 | 0 | 0 | 0 | 1230 |
|  | 3 | 4335 | 0 | 96 | 35 | 0 | 4814 | 0 | 0 | 85 | 0 | 6353 | 0 | 0 | 0 | 0 | 2378 |
|  | 4 | 3524 | 0 | 72 | 23 | 0 | 3901 | 0 | 0 | 70 | 0 | 5147 | 0 | 0 | 1 | 0 | 1926 |
|  | 5 | 2412 | 0 | 33 | 19 | 0 | 2656 | 0 | 0 | 46 | 0 | 3506 | 0 | 0 | 0 | 0 | 1312 |
|  | 6 | 2926 | 0 | 49 | 28 | 0 | 3237 | 0 | 0 | 69 | 0 | 4260 | 0 | 1 | 3 | 0 | 1595 |
|  | 7 | 2689 | 0 | 51 | 32 | 0 | 2988 | 0 | 0 | 66 | 0 | 3930 | 0 | 1 | 0 | 0 | 1475 |
|  | 8 | 1418 | 0 | 28 | 17 | 0 | 1576 | 0 | 1 | 35 | 0 | 2074 | 0 | 0 | 0 | 0 | 779 |
|  | 9 | 2623 | 0 | 45 | 27 | 0 | 2905 | 0 | 0 | 63 | 0 | 3822 | 0 | 0 | 1 | 0 | 1434 |
| **2** | 1 | 2882 | 0 | 33 | 11 | 0 | 3078 | 0 | 0 | 32 | 0 | 4148 | 0 | 0 | 12 | 0 | 1660 |
|  | 2 | 6594 | 0 | 105 | 0 | 0 | 7047 | 0 | 0 | 77 | 0 | 9465 | 28 | 0 | 37 | 0 | 3791 |
|  | 3 | 2802 | 0 | 24 | 23 | 0 | 2997 | 0 | 0 | 36 | 0 | 4029 | 5 | 3 | 0 | 0 | 1625 |
|  | 4 | 2676 | 0 | 19 | 0 | 1 | 2834 | 0 | 0 | 40 | 0 | 3801 | 9 | 8 | 4 | 0 | 1528 |
|  | 5 | 2743 | 0 | 28 | 1 | 2 | 2912 | 0 | 2 | 41 | 1 | 3896 | 22 | 2 | 1 | 0 | 1581 |
|  | 6 | 4578 | 0 | 42 | 0 | 2 | 4855 | 0 | 3 | 77 | 13 | 6487 | 23 | 1 | 2 | 0 | 2637 |
|  | 7 | 6837 | 0 | 90 | 3 | 1 | 7289 | 0 | 0 | 99 | 32 | 9732 | 37 | 0 | 3 | 0 | 3957 |
|  | 8 | 7039 | 47 | 152 | 0 | 33 | 7545 | 21 | 15 | 159 | 53 | 10062 | 66 | 2 | 43 | 12 | 4079 |
|  | 9 | 7599 | 0 | 101 | 0 | 10 | 8090 | 0 | 0 | 151 | 18 | 10795 | 36 | 3 | 7 | 0 | 4390 |
|  | 10 | 4536 | 0 | 84 | 0 | 0 | 4859 | 0 | 1 | 73 | 21 | 6474 | 32 | 0 | 0 | 0 | 2640 |
|  | 11 | 2025 | 0 | 39 | 15 | 0 | 2187 | 0 | 0 | 29 | 11 | 2906 | 24 | 0 | 0 | 0 | 1188 |
| **3** | 1 | 2991 | 0 | 51 | 0 | 0 | 3197 | 0 | 1 | 11 | 0 | 4240 | 0 | 2 | 0 | 2 | 1673 |
|  | 2 | 3592 | 0 | 74 | 0 | 0 | 3854 | 0 | 0 | 17 | 0 | 5106 | 0 | 2 | 1 | 6 | 2012 |
|  | 3 | 3008 | 0 | 112 | 0 | 34 | 3246 | 0 | 0 | 74 | 0 | 4286 | 0 | 1 | 4 | 0 | 1715 |
|  | 4 | 8006 | 0 | 260 | 2 | 58 | 8616 | 0 | 18 | 140 | 0 | 11414 | 0 | 55 | 68 | 0 | 4435 |
|  | 5 | 7796 | 0 | 315 | 1 | 86 | 8365 | 0 | 77 | 118 | 1 | 11217 | 0 | 99 | 124 | 0 | 4249 |
|  | 6 | 4274 | 0 | 172 | 0 | 94 | 4561 | 0 | 19 | 97 | 0 | 6116 | 0 | 55 | 32 | 0 | 2364 |
|  | 7 | 3915 | 0 | 141 | 0 | 89 | 4162 | 0 | 13 | 89 | 0 | 5579 | 0 | 54 | 19 | 0 | 2163 |
|  | 8 | 2473 | 0 | 97 | 4 | 66 | 2638 | 0 | 2 | 72 | 0 | 3525 | 0 | 34 | 4 | 0 | 1381 |
|  | 9 | 2545 | 1 | 105 | 1 | 67 | 2713 | 2 | 6 | 81 | 4 | 3619 | 2 | 34 | 8 | 0 | 1420 |
| **Pt** | **Sample** | 1. **Complete region without the editing codon** | | | | | | | | | | | | | | | |
|  |  | AA | AC | AG | AT | CA | CC | CG | CT | GA | GC | GG | GT | TA | TC | TG | TT |
| **1** | 1 | 2933 | 0 | 18 | 13 | 0 | 3237 | 0 | 0 | 34 | 0 | 4256 | 0 | 0 | 0 | 0 | 1560 |
|  | 2 | 2256 | 1 | 12 | 11 | 1 | 2486 | 2 | 1 | 44 | 2 | 3253 | 1 | 0 | 0 | 0 | 1200 |
|  | 3 | 4302 | 0 | 71 | 35 | 0 | 4814 | 0 | 0 | 85 | 0 | 6295 | 0 | 0 | 0 | 0 | 2320 |
|  | 4 | 3493 | 0 | 56 | 23 | 0 | 3901 | 0 | 0 | 70 | 0 | 5100 | 0 | 0 | 1 | 0 | 1879 |
|  | 5 | 2393 | 0 | 20 | 19 | 0 | 2656 | 0 | 0 | 46 | 0 | 3474 | 0 | 0 | 0 | 0 | 1280 |
|  | 6 | 2902 | 0 | 34 | 28 | 0 | 3237 | 0 | 0 | 69 | 0 | 4221 | 0 | 1 | 3 | 0 | 1556 |
|  | 7 | 2666 | 0 | 38 | 32 | 0 | 2988 | 0 | 0 | 66 | 0 | 3894 | 0 | 1 | 0 | 0 | 1439 |
|  | 8 | 1406 | 0 | 21 | 17 | 0 | 1576 | 0 | 1 | 35 | 0 | 2055 | 0 | 0 | 0 | 0 | 760 |
|  | 9 | 2602 | 0 | 31 | 27 | 0 | 2905 | 0 | 0 | 63 | 0 | 3787 | 0 | 0 | 1 | 0 | 1399 |
| **2** | 1 | 2862 | 0 | 15 | 11 | 0 | 3078 | 0 | 0 | 32 | 0 | 4110 | 0 | 0 | 12 | 0 | 1622 |
|  | 2 | 6546 | 0 | 66 | 0 | 0 | 7047 | 0 | 0 | 77 | 0 | 9378 | 28 | 0 | 37 | 0 | 3704 |
|  | 3 | 2780 | 0 | 9 | 23 | 0 | 2997 | 0 | 0 | 36 | 0 | 3992 | 5 | 3 | 0 | 0 | 1588 |
|  | 4 | 2653 | 0 | 7 | 0 | 1 | 2834 | 0 | 0 | 40 | 0 | 3766 | 9 | 8 | 4 | 0 | 1493 |
|  | 5 | 2723 | 0 | 12 | 1 | 2 | 2912 | 0 | 2 | 41 | 1 | 3860 | 22 | 2 | 1 | 0 | 1545 |
|  | 6 | 4543 | 0 | 17 | 0 | 2 | 4855 | 0 | 3 | 77 | 13 | 6427 | 23 | 1 | 2 | 0 | 2577 |
|  | 7 | 6783 | 0 | 54 | 3 | 1 | 7289 | 0 | 0 | 99 | 32 | 9642 | 37 | 0 | 3 | 0 | 3867 |
|  | 8 | 6985 | 47 | 112 | 0 | 33 | 7545 | 21 | 15 | 159 | 53 | 9968 | 66 | 2 | 43 | 12 | 3985 |
|  | 9 | 7531 | 0 | 69 | 0 | 10 | 8090 | 0 | 0 | 151 | 18 | 10695 | 36 | 3 | 7 | 0 | 4290 |
|  | 10 | 4504 | 0 | 56 | 0 | 0 | 4859 | 0 | 1 | 73 | 21 | 6414 | 32 | 0 | 0 | 0 | 2580 |
|  | 11 | 2011 | 0 | 26 | 15 | 0 | 2187 | 0 | 0 | 29 | 11 | 2879 | 24 | 0 | 0 | 0 | 1161 |
| **3** | 1 | 2963 | 0 | 40 | 0 | 0 | 3197 | 0 | 1 | 11 | 0 | 4201 | 0 | 2 | 0 | 2 | 1634 |
|  | 2 | 3564 | 0 | 55 | 0 | 0 | 3854 | 0 | 0 | 17 | 0 | 5059 | 0 | 2 | 1 | 6 | 1965 |
|  | 3 | 2984 | 0 | 96 | 0 | 34 | 3246 | 0 | 0 | 74 | 0 | 4246 | 0 | 1 | 4 | 0 | 1675 |
|  | 4 | 7977 | 0 | 183 | 2 | 58 | 8616 | 0 | 18 | 79 | 0 | 11369 | 0 | 55 | 68 | 0 | 4329 |
|  | 5 | 7779 | 0 | 228 | 1 | 86 | 8365 | 0 | 77 | 42 | 1 | 11189 | 0 | 99 | 124 | 0 | 4145 |
|  | 6 | 4251 | 0 | 138 | 0 | 94 | 4561 | 0 | 19 | 80 | 0 | 6076 | 0 | 55 | 32 | 0 | 2307 |
|  | 7 | 3890 | 0 | 114 | 0 | 89 | 4162 | 0 | 13 | 79 | 0 | 5537 | 0 | 54 | 19 | 0 | 2111 |
|  | 8 | 2457 | 0 | 80 | 4 | 66 | 2638 | 0 | 2 | 72 | 0 | 3492 | 0 | 34 | 4 | 0 | 1348 |
|  | 9 | 2526 | 1 | 90 | 1 | 67 | 2713 | 2 | 6 | 81 | 4 | 3585 | 2 | 34 | 8 | 0 | 1386 |

| **Pt** | **Sample** | **(c) Unedited genomes** | | | | | | | | | | | | | | | |
| --- | --- | --- | --- | --- | --- | --- | --- | --- | --- | --- | --- | --- | --- | --- | --- | --- | --- |
|  |  | AA | AC | AG | AT | CA | CC | CG | CT | GA | GC | GG | GT | TA | TC | TG | TT |
| **1** | 1 | 1879 | 0 | 13 | 8 | 0 | 2075 | 0 | 0 | 23 | 0 | 2727 | 0 | 0 | 0 | 0 | 1000 |
|  | 2 | 1276 | 0 | 7 | 9 | 0 | 1410 | 0 | 1 | 27 | 0 | 1843 | 0 | 0 | 0 | 0 | 680 |
|  | 3 | 2446 | 0 | 42 | 20 | 0 | 2739 | 0 | 0 | 50 | 0 | 3580 | 0 | 0 | 0 | 0 | 1320 |
|  | 4 | 2300 | 0 | 41 | 15 | 0 | 2573 | 0 | 0 | 47 | 0 | 3363 | 0 | 0 | 1 | 0 | 1239 |
|  | 5 | 1420 | 0 | 12 | 12 | 0 | 1577 | 0 | 0 | 30 | 0 | 2060 | 0 | 0 | 0 | 0 | 760 |
|  | 6 | 1784 | 0 | 22 | 18 | 0 | 1992 | 0 | 0 | 43 | 0 | 2597 | 0 | 1 | 2 | 0 | 957 |
|  | 7 | 1705 | 0 | 23 | 20 | 0 | 1909 | 0 | 0 | 42 | 0 | 2488 | 0 | 1 | 0 | 0 | 919 |
|  | 8 | 890 | 0 | 12 | 10 | 0 | 995 | 0 | 1 | 23 | 0 | 1297 | 0 | 0 | 0 | 0 | 480 |
|  | 9 | 1560 | 0 | 19 | 17 | 0 | 1743 | 0 | 0 | 37 | 0 | 2273 | 0 | 0 | 0 | 0 | 840 |
| **2** | 1 | 1505 | 0 | 8 | 7 | 0 | 1620 | 0 | 0 | 17 | 0 | 2163 | 0 | 0 | 4 | 0 | 856 |
|  | 2 | 3612 | 0 | 36 | 0 | 0 | 3888 | 0 | 0 | 43 | 0 | 5174 | 15 | 0 | 16 | 0 | 2048 |
|  | 3 | 1650 | 0 | 5 | 17 | 0 | 1782 | 0 | 0 | 21 | 0 | 2372 | 5 | 3 | 0 | 0 | 943 |
|  | 4 | 1744 | 0 | 4 | 0 | 1 | 1862 | 0 | 0 | 28 | 0 | 2472 | 7 | 4 | 1 | 0 | 984 |
|  | 5 | 1512 | 0 | 7 | 1 | 1 | 1619 | 0 | 0 | 22 | 0 | 2145 | 13 | 2 | 1 | 0 | 857 |
|  | 6 | 2650 | 0 | 10 | 0 | 1 | 2831 | 0 | 3 | 45 | 7 | 3749 | 14 | 1 | 1 | 0 | 1503 |
|  | 7 | 4066 | 0 | 35 | 3 | 0 | 4374 | 0 | 0 | 61 | 19 | 5782 | 24 | 0 | 0 | 0 | 2322 |
|  | 8 | 4024 | 20 | 60 | 0 | 12 | 4347 | 9 | 6 | 82 | 26 | 5740 | 38 | 2 | 21 | 6 | 2293 |
|  | 9 | 5119 | 0 | 49 | 0 | 9 | 5499 | 0 | 0 | 103 | 17 | 7269 | 23 | 3 | 5 | 0 | 2916 |
|  | 10 | 2399 | 0 | 33 | 0 | 0 | 2591 | 0 | 1 | 37 | 10 | 3424 | 17 | 0 | 0 | 0 | 1376 |
|  | 11 | 1046 | 0 | 13 | 5 | 0 | 1134 | 0 | 0 | 16 | 8 | 1490 | 12 | 0 | 0 | 0 | 602 |
| **3** | 1 | 2130 | 0 | 30 | 0 | 0 | 2300 | 0 | 1 | 8 | 0 | 3020 | 0 | 2 | 0 | 1 | 1170 |
|  | 2 | 2120 | 0 | 32 | 0 | 0 | 2300 | 0 | 0 | 11 | 0 | 3010 | 0 | 1 | 1 | 3 | 1170 |
|  | 3 | 1790 | 0 | 57 | 0 | 20 | 1950 | 0 | 0 | 44 | 0 | 2550 | 0 | 0 | 2 | 0 | 1010 |
|  | 4 | 6770 | 0 | 157 | 2 | 48 | 7320 | 0 | 14 | 63 | 0 | 9660 | 0 | 50 | 65 | 0 | 3660 |
|  | 5 | 6950 | 0 | 211 | 1 | 78 | 7480 | 0 | 69 | 37 | 1 | 10000 | 0 | 87 | 115 | 0 | 3700 |
|  | 6 | 2980 | 0 | 100 | 0 | 65 | 3200 | 0 | 16 | 49 | 0 | 4270 | 0 | 37 | 27 | 0 | 1620 |
|  | 7 | 2610 | 0 | 81 | 0 | 61 | 2800 | 0 | 9 | 53 | 0 | 3730 | 0 | 35 | 14 | 0 | 1420 |
|  | 8 | 1190 | 0 | 37 | 2 | 32 | 1280 | 0 | 1 | 35 | 0 | 1690 | 0 | 17 | 2 | 0 | 653 |
|  | 9 | 1410 | 0 | 49 | 1 | 37 | 1520 | 1 | 2 | 43 | 2 | 2010 | 0 | 19 | 4 | 0 | 775 |

| **Pt** | **Sample** | 1. **Edited genomes** | | | | | | | | | | | | | | | |
| --- | --- | --- | --- | --- | --- | --- | --- | --- | --- | --- | --- | --- | --- | --- | --- | --- | --- |
|  |  | AA | AC | AG | AT | CA | CC | CG | CT | GA | GC | GG | GT | TA | TC | TG | TT |
| **1** | 1 | 1054 | 0 | 5 | 5 | 0 | 1162 | 0 | 0 | 11 | 0 | 1529 | 0 | 0 | 0 | 0 | 560 |
|  | 2 | 980 | 1 | 5 | 2 | 1 | 1076 | 2 | 0 | 17 | 2 | 1410 | 1 | 0 | 0 | 0 | 520 |
|  | 3 | 1856 | 0 | 29 | 15 | 0 | 2075 | 0 | 0 | 35 | 0 | 2715 | 0 | 0 | 0 | 0 | 1000 |
|  | 4 | 1193 | 0 | 15 | 8 | 0 | 1328 | 0 | 0 | 23 | 0 | 1737 | 0 | 0 | 0 | 0 | 640 |
|  | 5 | 973 | 0 | 8 | 7 | 0 | 1079 | 0 | 0 | 16 | 0 | 1414 | 0 | 0 | 0 | 0 | 520 |
|  | 6 | 1118 | 0 | 12 | 10 | 0 | 1245 | 0 | 0 | 26 | 0 | 1624 | 0 | 0 | 1 | 0 | 599 |
|  | 7 | 961 | 0 | 15 | 12 | 0 | 1079 | 0 | 0 | 24 | 0 | 1406 | 0 | 0 | 0 | 0 | 520 |
|  | 8 | 516 | 0 | 9 | 7 | 0 | 581 | 0 | 0 | 12 | 0 | 758 | 0 | 0 | 0 | 0 | 280 |
|  | 9 | 1042 | 0 | 12 | 10 | 0 | 1162 | 0 | 0 | 26 | 0 | 1514 | 0 | 0 | 1 | 0 | 559 |
| **2** | 1 | 1357 | 0 | 7 | 4 | 0 | 1458 | 0 | 0 | 15 | 0 | 1947 | 0 | 0 | 8 | 0 | 766 |
|  | 2 | 2934 | 0 | 30 | 0 | 0 | 3159 | 0 | 0 | 34 | 0 | 4204 | 13 | 0 | 21 | 0 | 1656 |
|  | 3 | 1130 | 0 | 4 | 6 | 0 | 1215 | 0 | 0 | 15 | 0 | 1620 | 0 | 0 | 0 | 0 | 645 |
|  | 4 | 909 | 0 | 3 | 0 | 0 | 972 | 0 | 0 | 12 | 0 | 1294 | 2 | 4 | 3 | 0 | 509 |
|  | 5 | 1211 | 0 | 5 | 0 | 1 | 1293 | 0 | 2 | 19 | 1 | 1715 | 9 | 0 | 0 | 0 | 688 |
|  | 6 | 1893 | 0 | 7 | 0 | 1 | 2024 | 0 | 0 | 32 | 6 | 2678 | 9 | 0 | 1 | 0 | 1074 |
|  | 7 | 2717 | 0 | 19 | 0 | 1 | 2915 | 0 | 0 | 38 | 13 | 3860 | 13 | 0 | 3 | 0 | 1545 |
|  | 8 | 2961 | 27 | 52 | 0 | 21 | 3198 | 12 | 9 | 77 | 27 | 4228 | 28 | 0 | 22 | 6 | 1692 |
|  | 9 | 2412 | 0 | 20 | 0 | 1 | 2591 | 0 | 0 | 48 | 1 | 3426 | 13 | 0 | 2 | 0 | 1374 |
|  | 10 | 2105 | 0 | 23 | 0 | 0 | 2268 | 0 | 0 | 36 | 11 | 2990 | 15 | 0 | 0 | 0 | 1204 |
|  | 11 | 965 | 0 | 13 | 10 | 0 | 1053 | 0 | 0 | 13 | 3 | 1389 | 12 | 0 | 0 | 0 | 559 |
| **3** | 1 | 837 | 0 | 10 | 0 | 0 | 902 | 0 | 0 | 3 | 0 | 1185 | 0 | 0 | 0 | 1 | 461 |
|  | 2 | 1440 | 0 | 23 | 0 | 0 | 1558 | 0 | 0 | 6 | 0 | 2046 | 0 | 1 | 0 | 3 | 794 |
|  | 3 | 1193 | 0 | 39 | 0 | 14 | 1298 | 0 | 0 | 30 | 0 | 1698 | 0 | 1 | 2 | 0 | 669 |
|  | 4 | 1206 | 0 | 26 | 0 | 10 | 1298 | 0 | 4 | 16 | 0 | 1712 | 0 | 5 | 3 | 0 | 664 |
|  | 5 | 830 | 0 | 17 | 0 | 8 | 886 | 0 | 8 | 5 | 0 | 1183 | 0 | 12 | 9 | 0 | 441 |
|  | 6 | 1271 | 0 | 38 | 0 | 29 | 1362 | 0 | 3 | 31 | 0 | 1805 | 0 | 18 | 5 | 0 | 691 |
|  | 7 | 1276 | 0 | 33 | 0 | 28 | 1362 | 0 | 4 | 26 | 0 | 1810 | 0 | 19 | 5 | 0 | 690 |
|  | 8 | 1264 | 0 | 43 | 2 | 34 | 1359 | 0 | 1 | 37 | 0 | 1799 | 0 | 17 | 2 | 0 | 695 |
|  | 9 | 1113 | 1 | 41 | 0 | 30 | 1195 | 1 | 4 | 38 | 2 | 1578 | 2 | 15 | 4 | 0 | 611 |
